# Glycosaminoglycan Monosaccharide Blocks Analysis by Quantum Mechanics, Molecular Dynamics, and Nuclear Magnetic Resonance

**DOI:** 10.1155/2014/808071

**Published:** 2014-04-07

**Authors:** Sergey A. Samsonov, Stephan Theisgen, Thomas Riemer, Daniel Huster, M. Teresa Pisabarro

**Affiliations:** ^1^Structural Bioinformatics, BIOTEC, TU Dresden, Tatzberg 47-51, 01307 Dresden, Germany; ^2^Institute of Medical Physics and Biophysics, University of Leipzig, Härtelstr. 16-18, 04107 Leipzig, Germany

## Abstract

Glycosaminoglycans (GAGs) play an important role in many biological processes in the extracellular matrix. In a theoretical approach, structures of monosaccharide building blocks of natural GAGs and their sulfated derivatives were optimized by a B3LYP6311ppdd//B3LYP/6-31+G(d) method. The dependence of the observed conformational properties on the applied methodology is described. NMR chemical shifts and proton-proton spin-spin coupling constants were calculated using the GIAO approach and analyzed in terms of the method's accuracy and sensitivity towards the influence of sulfation, O1-methylation, conformations of sugar ring, and **ω** dihedral angle. The net sulfation of the monosaccharides was found to be correlated with the ^1^H chemical shifts in the methyl group of the N-acetylated saccharides both theoretically and experimentally. The **ω** dihedral angle conformation populations of free monosaccharides and monosaccharide blocks within polymeric GAG molecules were calculated by a molecular dynamics approach using the GLYCAM06 force field and compared with the available NMR and quantum mechanical data. Qualitative trends for the impact of sulfation and ring conformation on the chemical shifts and proton-proton spin-spin coupling constants were obtained and discussed in terms of the potential and limitations of the computational methodology used to be complementary to NMR experiments and to assist in experimental data assignment.

## 1. Introduction

Glycosaminoglycans (GAGs) represent a class of linear anionic heteropolysaccharides containing repeating disaccharide units made up of a hexose or a hexuronic acid linked to a hexosamine by a 1-3 or 1-4 glycosidic linkage. Hydroxyl groups of these saccharides can be sulfated at different positions. Being localized in the extracellular matrix, GAGs play a crucial role in cell adhesion and proliferation [1] by involvement in key molecular regulatory mechanisms [2]. As for all saccharides, GAGs are very flexible and adopt a number of energetically similar conformational states under physiological conditions, which render structural studies of GAGs challenging from both the experimental [3, 4] and the computational [5] points of view. Solvent is suggested to play an indispensable role for the structure and dynamics of saccharides due to the tight coupling of solvent and solute dynamics, their interactions [6–10], and the effects of electrostatic polarization [11]. In addition, the highly charged nature of GAGs makes their interactions with solvent molecules by hydrogen bonding even more important for the exploration of their conformational space [10, 12–15]. The rapid exchange of the intramolecular and solvent-mediated hydrogen bonds does not allow experimental techniques such as nuclear magnetic resonance (NMR) to gain a deep view on the hydrogen bonds formation in GAGs and, therefore, computational methods as molecular dynamics (MD) simulations are very useful to analyze GAGs structural properties in more detail [16]. Regarding the sulfation patterns of GAGs, combination of NMR with MD [17] and quantum mechanical (QM) [18] approaches were successfully applied to reveal the impact of sulfation effects on GAGs structure in terms of dynamic behaviour of glycosidic linkages. However, not only glycosidic linkage conformations but also sugar ring puckering could be decisive for the biologic relevance and the specificity of GAG/protein interactions [19]. In the case of heparin, it is supposed that the conformational flexibility of the free heparin molecule is not dramatically affected by the flexibility of the IdoUA(2S) sugar rings [20]. Nevertheless, it was reported that in the complex of heparin pentasaccharide with FGFR one of the IdoUA(2S) adopted the ^2^S_0_ ring conformation, whereas the rest of IdoUA(2S) residues were in the ^1^C_4_ ring conformation, providing high specificity for the formation of this GAG/protein complex [21]. Therefore, it is important to understand the basic rules governing the ring conformation preferences for individual monosaccharide blocks of the GAG molecules. The ring conformational space for several GAG mono- and disaccharides for GlcNAc and its N-, 3-O, and 6-O sulfated derivatives [22], GlcUA, IdoUA [23, 24], IdoUA(2S) [23, 25, 26], and heparin disaccharides [27, 28], was extensively analyzed in recent studies by means of MD, QM, and NMR approaches, demonstrating agreement and complementarity of these methodologies. This suggests a high potential of the use of theoretical approaches for the assistance in interpretation of NMR experimental data. Interestingly, despite the above-mentioned important role of solvent, the use of an implicit solvent model (in contrast to the use of explicit solvent molecules) does not improve agreement between spin-spin coupling parameters calculated by QM and measured experimentally by NMR [26].

In addition to natural GAGs, artificial GAGs with distinct sulfation patterns are promising components for functional biomaterials targeted for extracellular artificial matrix engineering since additional sulfate groups could modulate specific binding of growth factors and thereby influence wound healing [29–31]. Unfortunately, sometimes only the net sulfation degree of GAGs used in the experiments but not the exact sulfation pattern is known, which renders assignment of NMR spectra for the following elucidation of structure-function relationships more challenging. Therefore, theoretical analysis of the structural properties of sulfated GAG monosaccharides and calculation of their NMR chemical shifts and spin-spin coupling constants could be essential for the assistance in NMR experimental data interpretation. Along these lines, it was shown that specific sulfation patterns of some GlcNAc derivatives induce changes in ring puckering preferences [22]. Here, we systematically study sulfated derivatives of GlcNAc, GalNAc, IdoUA, and GlcUA with varying degrees of net sulfation, which represent the building blocks for heparin, hyaluronic acid, chondroitin sulfate, and dermatan sulfate. In particular, we analyze conformational preferences of the sugar rings using a QM approach, the impact of sulfation, and used polymerization models. Furthermore, we calculate NMR parameters using several computational models, which provide GAG monosaccharide conformational QM dictionary data. For GlcNAc, GlcNAc(6S), GalNAc, GalNAc(4S), and GalNAc(6S), we compare our calculated parameters with experimental data on ^13^C and ^1^H chemical shifts and proton-proton spin-spin coupling constants (^3^J_H-H_), and we discuss the potential accuracy of this methodology. For GlcNAc and GalNAc sulfated derivatives, the conformational space of the dihedral angle around the C5–C6 bond is analyzed and compared within QM and MD approaches. The data obtained in this work help to get a deeper insight in the potential and limitations of state-of-the-art computational methods used to complement NMR experiment interpretation for GAG molecules.

## 2. Materials and Methods

### 2.1. Quantum Mechanical Calculation

**Figure 1:**
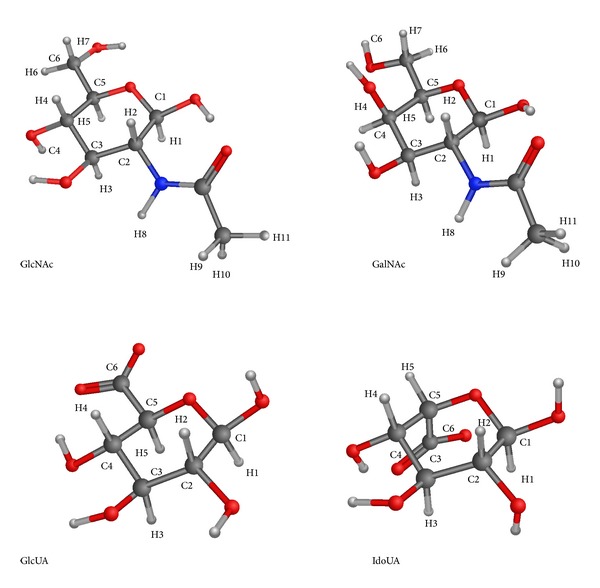
Chemical structure of**β**-D-GlcNAc,**β**-D-GalNAc,**β**-D-GlcUA, and**α**-L-IdoUA with numbering used throughout the paper.

The following monosaccharides (Figure 1) and their* O*1-methylated variants (abbreviated with M- in Tables 1 and 2) were used for QM calculations: GlcNAc (**β**-D-N-acetylglucosamine), GlcNAc(4S) (4-*O*-sulfo-**β**-D-N-acetylglucosamine), GlcNAc(6S) (6-*O*-sulfo-**β**-D-N-acetylglucosamine), GlcNAc(46S) (4,6-*O*-disulfo-**β**-D-N-acetylglucosamine), GalNAc (**β**-D-N-acetylgalactosamine), GalNAc(4S) (4-*O*-sulfo-**β**-D-N-acetylgalactosamine), GalNAc(6S) (6-*O*-sulfo-**β**-D-N-acetylgalactosamine), GalNAc(46S) (4,6-*O*-disulfo-**β**-D-N-acetylgalactosamine), GlcUA (*β*-D-glucuronic acid), GlcUA(2S) (2-*O*-sulfo-*β*-D-glucuronic acid), GlcUA(3S) (3-*O*-sulfo-*β*-D-glucuronic acid), GlcUA(23S) (2,3-*O*-disulfo-*β*-D-glucuronic acid), IdoUA (**α**-L-iduronic acid), IdoUA(2S) (2-*O*-sulfo-**α**-L-iduronic acid), and IdoUA(3S) (3-*O*-sulfo-**α**-L-iduronic acid), IdoUA(23S) (2,3-*O*-disulfo-**α**-L-iduronic acid).

First, the molecules were built in MOE [32] in ^1^C_4_, ^4^C_1_, and ^2^S_0_ ring conformations. For Glc/GalNAc sulfated derivatives* gt*,* tg,* and* gg* conformations were built corresponding to the values of dihedral angle *ω* = (O6–C6–C5–O5) of ~300°, ~60°, and ~180°, respectively [10]. Na^+^ counterions were manually added to the systems with a nonzero net charge, and their positions were subsequently optimized by AMBER99 force field in MOE. The geometry optimization of these structures was carried out with GAUSSIAN 09 [33] using B3LYP functional [34] with 6-31+G(d) basis set. Single point energies were calculated using the B3LYP6311ppdd method, which was shown to be appropriate for energy calculations for carbohydrates [35]. For each monosaccharide, the relative energies were calculated using the energy of the most stable conformation as reference. GIAO methodology implemented within GAUSSIAN [36] was used to calculate NMR parameters: B3LYP6311+G(2d,p) for chemical shifts and B3LYP/aug-cc-pVDZ for spin-spin coupling constants, as these levels of theory demonstrated highest reliability in the calibration studies [37, 38]. TMS (tetramethylsilane) was used as a reference to calculate ^13^C- and ^1^H-chemical shifts. For the calculations carried out in solvent, PCM solvent model [39] was used.

### 2.2. Molecular Dynamics Calculations

For MD simulations, the GLYCAM06 force field [40] implemented in the AMBER 11 package [41] was used for GAGs. For sulfated residues, sulfate atomic charges for HA and CS derivatives residue libraries were obtained from RESP by fitting calculations at the level of 631(d)G for methylsulfate and introduced into the corresponding GLYCAM libraries. All the monosaccharides were modeled in the ^4^C_1_ ring conformation as it was suggested by our results (see Section 3.1). Prior to the simulation, GAG monosaccharides were solvated within an octahedral TIP3PBOX of 15 Å distance to the sides of the periodic unit, and counterions were added when required. The system was minimized and equilibrated as described before [42] and simulated for 50 ns in NTP ensemble. For MD simulations of GAGs hexasaccharides, the structures available in the PDB for octameric HA (PDB ID: 2BVK, NMR) and hexameric CS4 (PDB ID: 1CS4, fiber diffraction) were used as templates for modeling of the following HA and CS derivatives: (GlcUA-GlcNAc)_3_, (GlcUA-GlcNAc(4S))_3_, (GlcUA-GlcNAc(6S))_3_, (GlcUA-GlcNAc(46S))_3_, (GlcUA(2S)-GlcNAc(46S))_3_, (GlcUA(3S)-GlcNAc(46S))_3_, (GlcUA(23S)-GlcNAc(46S))_3_, (GlcUA-GalNAc)_3_, (GlcUA-GalNAc(4S))_3_, (GlcUA-GalNAc(6S))_3_, (GlcUA-GalNAc(46S))_3_, (GlcUA(2S)-GalNAc(46S))_3_, (GlcUA(3S)-GalNAc(46S))_3_, and (GlcUA(23S)-GalNAc(46S))_3_. The MD simulations for these GAGs were carried out for 20 ns, and the obtained data for three monosaccharide units within each hexasaccharide were averaged to be compared with the data on free monosaccharides. The trajectories analysis was done using the ptraj module of AMBER 11. For the analysis of the dihedral angle *ω* = (O6–C6–C5–O5) for Glc/GalNAc sulfated derivatives,* gg*,* gt*, and* tg*, conformations were defined for *ω* in the ranges of [−120°; 0°), [0°; 120°), and [−180°; −120°) ∪ [120°; 180°), respectively.

### 2.3. NMR Measurements

All NMR spectra were measured on a Bruker Avance III 600 MHz spectrometer operating at 600.13 MHz ^1^H resonance frequency equipped with a 5 mm TBI triple resonance probehead with Z-gradient or on a Bruker Avance I 700 MHz spectrometer operating at 700.18 MHz ^1^H resonance frequency equipped with a triple resonance cryo-probehead at 37°C in D_2_O with TSP as a reference (set to 0 ppm for ^1^H and ^13^C chemical shifts). The resonance assignments were based on COSY, J-modulated, and HSQC 2D spectra. To account for strong coupling effects, the chemical shifts and ^3^J_H-H_ were extracted by fitting the experimental 1D spectra with a self-written Octave script [43].

Statistical analysis of data was carried out with the R-package [44].

## 3. Results and Discussion

### 3.1. Conformational Preferences of the Analyzed Monosaccharides

The geometries of GlcNAc, GalNAc, GlcUA, and IdoUA monosaccharides and their sulfated derivatives were optimized and their single point energies were calculated for three ring conformations (^4^C_1_, ^1^C_4_, ^2^S_0_) and, in addition, for the* gg*/*gt*/*tg* conformations for N-acetylated saccharides (Tables 1 and 2). The obtained results represent* in vacuo *and PCM implicit solvent models for nonmethylated and* O*1-methylated monosaccharides, where the latter is used as the simplest model for the glycosidic linkage in GAGs. Using the same level of theory for single point energy calculations (B3LYP6311ppdd) but a different level for geometry optimization (6-31+G(d) versus B3LYP6311ppdd), we were able to nicely reproduce relative energies for M-IdoUA(2S) ring conformers obtained in the work of Hricovíni [26] (7.690 versus 7.18; 2.775 versus 2.77 kcal/mol, for the differences between the most stable ^1^C_4_ and ^2^S_0_; ^4^C_1_ conformations, resp.). The positions of counterions were also predicted very similarly to the positions in the aforementioned study. If the counterions were not used for the calculations, though the geometry of M-IdoUA(2S) was correctly obtained, energetic comparison of the conformations failed. For example, when not using counterions, the ^4^C_1_ ring conformation was observed to be the most stable (data not shown). This suggests a strong impact of the ions on the ring puckering due to the net electrostatic effect in a not neutralized system. Interestingly, final point energies are also affected by the counterion positions occupied after the geometry minimization. In case of many negatively charged groups as, for example, for double sulfated GlcUA or IdoUA, these positions could be not unique. This point is important to consider when quantitatively analyzing the results represented in Tables 1 and 2.

For GlcNAc and GalNAc derivatives, all the data show the preference for the ^4^C_1_ ring conformation (except for M-GlcNAc* in vacuo*, where ^2^S_0_ was found to be the most stable with a relatively low difference of 1.09 kcal/mol to the ^4^C_1_ conformation) (Table 2). This agrees with the previous long MD studies for GlcNAc [22] and the experimental structures of free chondroitin sulfate 4 (PDB ID: 1CS4) and hyaluronic acid (PDB IDs: 1HYA, 2HYA, 3HYA, 4HYA, 1HUA, 2BVK). In general, the probability of adopting ^2^S_0_ was calculated to be higher than for ^1^C_4_ for the optimized structures. When analyzing* gg*/*gt*/*tg* conformations of *ω* dihedral angle, there is an essential dependence on the model used for the calculations. Nevertheless, for both* in vacuo* and implicit solvent calculations, GlcNAc derivatives prefer* tg* conformation, while GalNAc derivatives are more prone to the* gg* conformation, which is not in agreement with the expected gauche effect for these molecules. This inconsistency of QM methods was also observed previously in the work of Kirschner and Woods. These authors explain the limitation of this methodology in terms of disregarding explicit solvent [10]. Indeed, the* tg* conformation for structure of GlcNAc obtained by QM is favourable due to the formation of a hydrogen bond between O6 and HO4 atoms, which in the presence of explicit solvent could be disrupted due to the interaction with water molecules. In case of GlcNAc(4S) and GlcNAc(6S), the strong interaction between sulfate groups and hydrogens of the hydroxyl groups defines the most favourable *ω* dihedral angle conformation. Besides that, the positions of counterions are especially important: for GlcNAc(46S) two Na^+^ ions are coordinated between sulfate groups in the positions 4 and 6 and, therefore, strongly stabilize the* tg* conformation. For GalNAc, the formation of the hydrogen bond between O6 and HO4 atoms is more probable in the* gg* conformation. For the sulfated GalNAc derivatives, the positions of Na^+^ ions are crucial for the selection of the most energetically favourable *ω* dihedral angle conformation. Methylation of the O1 decreases the opportunity for intramolecular hydrogen bonding and, therefore, also influences the* gg*/*gt*/*tg* conformational distribution.

For GlcUA sulfated derivatives, the conformational dependence on both sulfation pattern and the model used for calculations is clearly observed (Table 2). For GlcUA monosaccharide, all methods find the ^4^C_1_ ring conformation highly probable.* In vacuo* calculations propose coexistence of this conformation together with the ^1^C_4_ conformation with the prevalence of the latter for the case when O1 position is not methylated (difference in energy of two conformations of 1 kcal/mol corresponds to their probabilities ratio of 85 : 15). This agrees with the experimental structures of free hyaluronic acid (PDB IDs: 1HYA, 2HYA, 3HYA, 4HYA, 1HUA, 2BVK) and data from MD simulations [23]. For sulfated derivatives of GlcUA, all* O*1-methylated monosaccharides are found to be the most stable in the ^4^C_1_ conformation independently of the solvent use in the calculations. For M-GlcUA(3S), the differences in energies between ^2^S_0_ and ^4^C_1_ are quite low suggesting possible coexistence of these conformations. For all unmethylated sulfated derivatives of GlcUA (except for* in vacuo* calculation for GlcUA(3S), where the ^1^C_4_ conformation was the most stable), the ^2^S_0_ conformation is preferred.

For IdoUA derivatives, there is a much higher consistency within the results obtained by different methods, though the relative differences between the conformations stabilities for different methods are still relatively high (e.g., IdoUA(3S)* in vacuo* versus implicit solvent) (Table 2). All the derivatives except IdoUA(2S) prefer the ^4^C_1_ conformation, whereas IdoUA(2S) prefers the ^1^C_4_ conformation. Interestingly, in the free heparin crystal structure (PDB ID: 1HPN), IdoUA(2S) monosaccharide units are observed in ^2^S_0_ and ^1^C_4_ but not in the ^4^C_1_ conformation.

All these QM data for the analyzed monosaccharides suggest that the results obtained for distinct models (with respect to solvent and O1-methylation) should be considered with caution, especially when compared to the data on conformational preferences for sugar rings and* gg*/*gt*/*tg* for these monosaccharides within long GAG polymers.

### 3.2. MD Conformational Analysis of *ω* Dihedral for Glc/GalNAc Derivatives

As it was pointed out in the previous section, QM approaches experience severe difficulties in the quantitative description of *ω* dihedral angle conformations. In contrast to QM calculations, MD simulations are able not only to take into account solvent explicitly but also to gain insights into internal motions of the molecules and, therefore, yield more complete information about the conformational space than the data from QM or NMR experiments.

According to the experimental data, glycopyranosyl derivatives tend to adopt* gg* and* gt* conformations (known as* gauche* effect) with the ratios of* gg*/*gt*/*tg* in the percentage range ~60–70 : 30–40 : 0–5 per conformation, whereas galactopyranosyl derivatives adopt less* gg* and gain in* tg* conformational content with the corresponding ratios of 10–20 : 45–55 : 30–40 [45–47]. MD simulations for GlcNAc and GalNAc nonmethylated derivatives in general agree with this trend and are able to reproduce the* gauche* effect (Table 3). For GlcNAc derivatives, sulfation in the 4th position increases the preference to the* gt *conformation, and sulfation in the 6th position makes* gg* more favourable. For GalNAc derivatives, sulfation in the 4th position does not make any significant effect, while sulfation in position 6 makes the* tg* conformation significantly more favourable. This could be explained in terms of the dipole interactions between the sulfate and hydroxyl groups in positions 4 and 6. For the data interpretation, several aspects should be taken into account: (i) the sulfation of the hydroxyl group changes the direction of the corresponding dipole to the opposite one; (ii) the absolute value of the OH group dipole is lower than the one of O–SO_3_; (iii) C4–O4 is in equatorial configuration for GlcNAc and in axial configuration for GalNAc; (iv) the flexibility of the group in position 6 is higher than the flexibility of the group in position 4; (v) the repulsive strength of the dipole-dipole interaction is defined by the dipole's absolute value, the distance between them, and their mutual orientation. With these considerations, the increase of the* gg* conformation population for GlcNAc(6S) in comparison to GlcNAc could be explained by more favourable interaction of O4–H dipole with O6–SO_3_ dipole in comparison to weak repulsion between two O–H dipoles in the nonsulfated derivative since the angle and distance between these dipoles are lower in* gg* in comparison to the* gt* conformation. On the contrary, the decrease of the* gg* conformation population of GlcNAc(46S) in comparison to GlcNAc(6S) could be explained by the stronger repulsion between two O4/O6–SO_3_ dipoles in the case of the GlcNAc(46S) molecule. For GalNAc derivatives, the* gg* conformation is sterically less accessible because the C4–O4 configuration is different to GlcNAc, and the O4–H group would overlap with the O6–SO_3_ group in this conformation. Here, the* tg* conformation is favourable when both O4 and O6 are sulfated because the angle between these two dipoles is closer to 90° than for the* gt* conformation. However, this explanation in terms of only two dipoles interaction cannot be used when comparing the differences in preferences of GalNAc(4S) and GalNAc(6S).

We also compared these data for monosaccharides MD simulations with the results obtained for the corresponding monosaccharide blocks within the hexameric GAGs (Table 4). When only the sulfation of GAGs changes, the populations of the *ω* dihedral angle conformation change very similarly to the ones in monosaccharides for HA, HA4, HA6, and HA46 and for CS_de, CS4, CS6, and CS46, respectively. Sulfation of the GlcUA within hexameric HA and CS derivatives affects the conformations of GlcNAc and GalNAc46 for GlcUA(2S) and GlcUA(3S), respectively. This suggests a pronounced mutual influence of electrostatic environment of the monosaccharide units within the polymer on their *ω* dihedral angle conformations but a weak influence of the polymerization via O1 and O3, respectively.

### 3.3. Chemical Shifts and ^3^ *J*_*H*-*H*_ Calculations

For ^13^C and ^1^H chemical shifts and ^3^J_H-H_ GIAO calculations, we used GlcNAc, GalNAc, GlcUA, and IdoUA monosaccharides and their sulfated derivatives, both nonmethylated and* O*1-methylated, in the conformations listed in Section 3.1. These data (see Supplementary Tables 1–17 available online at http://dx.doi.org/10.1155/2014/808071/) could be used as QM NMR parameters dictionary for monosaccharides with a different sulfation pattern.

Our analysis of ^13^C and ^1^H chemical shifts yields no significant dependence of these calculated NMR parameters neither on the conformations nor on O1-methylation (except for C1) (Supplementary Tables 1–12). In contrast, there are changes of chemical shifts occurring upon sulfation. In particular, ^13^C chemical shifts increase more than 5 ppm for C4 and slightly less than 5 ppm for C6 in case of GlcNAc and GalNAc sulfated monosaccharides. The same trend is observed in the experiments (Table 5), where the chemical shifts of the sulfated carbons C4 and C6 increased by 6 to 7 ppm. When C4 is sulfated, calculated chemical shifts of the adjacent C3 and C5 slightly drop, which is also observed in the experiments, where the similar chemical shift changes are observed for C5 when C6 is sulfated. In case of GlcUA and IdoUA sulfated derivatives, C2 and C3 chemical shifts similarly increase upon sulfation, while chemical shifts of adjacent carbons also drop. Our results allow for the conclusion that the calculated changes in the chemical shifts upon sulfation agree well with the experimental data. However, the variance within the values corresponding to different individual conformations is substantial. For example, for GlcUA ^1^C_4_ conformation, the increase of the chemical shift for C3 is observed upon the sulfation of C3, which contradicts the general observation derived from the averaging per all conformations. This makes the use of these values challenging for the practical purposes of the direct NMR spectra assignment. Based on the presented data, the expected error range for ^13^C chemical shifts is up to 5 ppm, which is similar to the differences found for the sulfated carbons. For the ^1^H chemical shifts, we also observe the significant increase of about 0.5 ppm for the values of the hydrogens bound to the carbons being sulfated as well as a slight increase of chemical shifts of the hydrogens bound to the carbons adjacent to the sulfated ones. Our experimental data for the C4-sulfation of GlcNAc and GalNAc qualitatively support the data obtained in our calculations (Table 5). For both ^13^C and ^1^H chemical shifts, we clearly observe experimentally validated qualitative trends, which might further allow for a quantitative comparison of QM obtained values with experimental data. The variance of the observed chemical shifts within the groups of different conformations is about 0.5 ppm and, therefore, comparable to the experimentally observed differences induced by sulfation.

For ^3^J_H-H_ we obtain slight qualitative differences depending on the sulfation pattern (Supplementary Tables 13–17), which is similarly observed by the experiment (Table 6). The variance of the ^3^J_H-H_ for the protons bound to the carbons C1–C5 of the ring is up to 2-3, whereas the variance of ^3^J_H-H_ for other proton pairs (available for GlcNAc and GalNAc derivatives) is higher and reaches the values of 4-5. The variance of the ^3^J_H-H_ grouped by ring conformations decreases down to 1 for the protons bound to the carbons C1–C5, which is similar to the corresponding experimental accuracy. In case of GlcNAc derivatives, especially high variance for the ^2^S_0_ ring conformation is observed. This is due to the fact that the geometry optimization starting from this ring conformation for M-GlcNAc(6S) and M-GlcNAc(46S) ended up in the ^4^C_1_ conformation, whereas for some molecules dramatic geometrical distortions were found (they correspond to high energies in Table 2). Except for GlcNAc derivatives in the ^2^S_0_ conformation, clear trends for ^3^J_H-H_ of H1-H2, H2-H3, H3-H4, and H4-H5 are observed, which allows significantly distinguishing different ring conformations within the applied method. In particular, GlcUA and IdoUA derivatives have four high ^3^J_H-H_ (~5–8) that correspond to the ^4^C_1_ conformation, four low (~2–4) to the ^1^C_4_ conformation, and three low and one high to the ^2^S_0_ conformation, respectively. For GlcNAc and GalNAc derivatives, epimeric C4 could be clearly distinguished for the corresponding H3-H4 and H4-H5 ^3^J_H-H_ for the most energetically favourable ^4^C_1_ conformation, which is qualitatively in agreement with the experimental data but quantitatively underestimated (Table 6). The values for* gg*/*gt*/*tg* conformations for each ring conformation clearly differ (Supplementary Tables 13–16), which corresponds to different geometries and could be used as a dictionary for these parameters. Therefore, calculations of ^3^J_H-H_ could be used for assistance in NMR assignments in cases where ring conformation and sulfation patterns are not well defined.

NMR parameters calculated by GIAO approaches (chemical shifts, ^3^J_H-H_) qualitatively reflect the sulfation, ring, and *ω* dihedral conformations (^3^J_H-H_). However, the direct and quantitative use of the calculated NMR parameters for experimental data assignment could be limited due to the intrinsic error of the method.

### 3.4. Comparison of the Calculated NMR Parameters with Experimental Data for GlcNAc, GlcNAc(6S), GalNAc, GalNAc(4S), and GalNAc(6S)

In order to estimate the practical applicability of the used computational methods for NMR parameter calculations, we compared the calculated chemical shifts and ^3^J_H-H_ with the available experimental data obtained by NMR for *β*-GlcNAc, *β*-GlcNAc(6S), *β*-GalNAc, *β*-GalNAc(4S), and *β*-GalNAc(6S) (Tables 5 and 6). The Pearson and Spearman correlations between the calculated and experimental data and the mean error of the theoretical methods are 0.994, 0.959, and 4.34 ppm for ^13^C chemical shifts, 0.961, 0.933, and 0.05 ppm for ^1^H chemical shifts, and 0.899, 0.840, and 1.3 Hz for ^3^J_H-H_, respectively (Table 7). For ^13^C chemical shifts, the theoretically obtained absolute values for all analyzed saccharides are systematically overestimated for C1–C7 except for C4 of GalNAc(4S) and underestimated for about 1 ppm for C8 (Table 5). The Pearson correlations between experimental and theoretical values are very high for ^13^C chemical shifts, which nevertheless could be partially explained in terms of high differences between the values for C8 in comparison to other values. Spearman correlations were found to be 1.0 for four out of five monosaccharides, which represents a promising result for ranking the peaks for ^13^C spectra. At the same time, the mean error could be too high for distinguishing carbons C1–6 in case the most probable conformation of the molecule is* a priori* unknown. For ^1^H chemical shifts, we obtained systematic underestimation of H3 and overestimation of H7 chemical shifts by the applied GIAO approach, while for other protons both overestimation and underestimation of experimental values were observed (Table 5). Both Pearson and Spearman correlations for ^1^H chemical shifts are lower than for ^13^C chemical shifts but the low mean error seems to be more promising for potential use of these chemical shifts for assisting NMR assignment. In addition, if more NMR data for the same class of molecules would be available, the mean and intercept of the linear regression between theoretical and experimental values could be used for scaling and inter/extrapolation of computational data in order to further minimize the mean error of the predicted values. For ^3^J_H-H_, the correlations are slightly lower and mean errors are higher. The values calculated by GIAO^3^J_H-H_ values for H1-H2, H2-H3 for all analyzed molecules and for H4-H5 for GlcNAc/GlcNAc(6S) are underestimated, while the corresponding ^3^J_H-H_ values for H4-H5 for GalNAc/GalNAc(6S) are overestimated (Table 6). For H3-H4, H5-H6, and H5-H7 both theoretical overestimation and underestimation in comparison to the experiment were obtained. Scaling of the calculated ^3^J_H-H_ based on the further obtained experimental data would assist the creation of a quantitative procedure to be used in NMR assignment for this class of molecules.

### 3.5. Sulfation Degree and Methyl-Group Chemical Shifts in Acetyl Group of Glc/GalNAc Derivatives

According to our computational data, despite the limitations for the chemical shifts calculations described above, we can clearly see a general increase of the H9/H10/H11 chemical shift value averaged for all* gg*/*tg*/*gt* conformations with an increase in the sulfation of the monosaccharides (Supplementary Table 18), whereas, for protons H10 and H11, there is only one significant increase of the chemical shift when a monosaccharide is sulfated once; the increase of the chemical shift value for proton H9 is significant in the order Glc/GalNAc, Glc/GalNAc(4S), Glc/GalNAc(6S), and Glc/GalNAc(46S) (Supplementary Table 19). These results show that, despite the expected moderate accuracy in the prediction of chemical shifts, the trend for such an important parameter as net sulfation of the monosaccharide being analyzed by the calculations of the methyl-group chemical shifts in the acetyl group of Glc/GalNAc derivatives agrees with the trend observed by NMR experimental data for the polymeric GAGs with different net sulfation degree.

## 4. Conclusions

In this work, we applied QM methodology in order to analyze the conformational space and NMR parameters (chemical shifts and ^3^J_H-H_) of GAG monosaccharide blocks. We investigated perspectives and limitations of the applicability of GIAO methodology for the assistance to NMR analysis of GAGs. We observed that in such conformational analysis the choice of the model for QM calculation has a significant impact on the results. Comparison of our QM and MD results for* gg*/*gt*/*tg* conformations distribution for GlcNAc and GalNAc stressed the importance of the use of explicit solvent for conformational analysis of saccharides by theoretical approaches. We found that calculated chemical shifts could be used for the analysis of the sulfation position of GAG monosaccharide blocks as well as the net sulfation, whereas ^3^J_H-H_ could be useful for both sulfation position and ring conformation analysis. Despite being promising, our results suggest that more experimental data are needed for optimization of the theoretically obtained parameters before being used to support NMR assignment.

## Supplementary Material

Supplementary Tables 1–12: The description should be "Chemical shifts calculated by GIAO approach".
Supplementary Tables 13–17: The description should be "Chemical shifts calculated by GIAO approach".
Supplementary Tables 13–16: The description should be "^3^J_H-H_ calculated by GIAO approach".
Supplementary Table 18: The description should be "^3^J_H-H_ calculated by GIAO approach".
Supplementary Table 19: 1H chemical shifts in CH_3_ of the acetyl group of Glc/GalNAc derivatives for gg/gt/tg conformations.
Supplementary Table 18: 1H chemical shifts in CH_3_ of the acetyl group of Glc/GalNAc derivatives.
Supplementary Table 19: 1H chemical shifts in CH_3_ of the acetyl group of Glc/GalNAc derivatives for gg/gt/tg conformations.

## Figures and Tables

**Table 1:** B3LYP6311ppdd//B3LYP/6-31+G(d) relative energies for ring and *gg/gt/tg* conformations of GlcNAc and GalNAc derivatives.

Molecule/conformation	Δ*E*(^4^C_1_), kcal/mol			Δ*E* (^1^C_4_), kcal/mol			Δ*E* (^2^S_0_), kcal/mol		
Saccharides	*gg *	*gt *	*tg *	*gg *	*gt *	*tg *	*gg *	*gt *	*tg *
GlcNAc	2.02	1.77	0	10.17	6.85	10.31	2.81	4.28	11.66
M-GlcNAc	3.40	2.72	1.09	5.67	2.01	6.04	1.67	0	7.40
GlcNAc_PCM_	0.39	0.28	0	7.83	6.39	8.29	4.04	4.25	9.65
M-GlcNAc_PCM_	4.02	2.55	0	3.11	4.28	6.97	4.81	3.02	8.22
GlcNAc(4S)	7.77	8.30	0	19.19	14.61	14.70	7.00	15.05	4.77
M-GlcNAc(4S)	16.46	13.76	0	19.37	15.11	15.10	1.71	15.94	8.22
GlcNAc(4S)_PCM_	3.31	3.31	0	10.04	9.15	8.38	3.98	7.33	4.38
M-GlcNAc(4S)_PCM_	5.26	2.83	0	8.61	6.61	6.44	4.59	5.65	6.28
GlcNAc(6S)	0.22	0	10.14	8.98	16.84	19.47	22.93	14.08	14.49
M-GlcNAc(6S)	6.94	3.45	0	1.30	12.49	11.76	15.04	11.53	16.06
GlcNAc(6S)_PCM_	0.27	0	1.52	7.18	5.28	6.83	8.51	4.62	6.06
M-GlcNAc(6S)_PCM_	0	0.35	2.46	2.67	2.66	2.56	≥^4^C_1_	4.1	5.79
GlcNAc(46S)	0	6.13	1.89	44.14	29.03	21.05	17.59	37.31	14.50
M-GlcNAc(46S)	11.96	7.90	0	23.16	24.90	12.29	≥^4^C_1_	28.70	0.49
GlcNAc(46S)_PCM_	1.61	3.78	0	12.45	9.27	8.28	9.44	11.52	10.78
M-GlcNAc(46S)_PCM_	3.71	4.65	0	8.86	8.89	4.56	7.33	8.32	1.75
GalNAc	0	2.03	3.67	9.29	8.88	12.10	0.78	6.98	10.95
M-GalNAc	0	2.07	2.81	3.99	3.60	5.95	3.43	4.51	4.09
GalNAc_PCM_	0	1.04	2.40	8.55	8.40	8.68	3.78	6.64	9.39
M-GalNAc_PCM_	0	1.99	3.70	5.74	5.50	6.11	7.36	6.77	6.98
GalNAc(4S)	7.77	8.30	0	19.19	14.61	14.70	7.00	15.05	4.77
M-GalNAc(4S)	4.99	0.80	0	10.43	26.57	15.24	7.22	24.22	7.79
GalNAc(4S)_PCM_	0	2.96	2.09	9.57	12.48	9.28	3.90	11.29	5.00
M-GalNAc(4S)_PCM_	0	0.31	3.09	7.25	11.13	8.31	6.21	8.59	4.45
GalNAc(6S)	0	4.69	15.80	10.42	20.96	20.91	23.99	20.26	21.75
M-GalNAc(6S)	3.14	0	5.86	11.79	11.01	5.44	12.10	10.36	10.23
GalNAc(6S)_PCM_	0	0.81	0.20	6.09	5.13	5.44	12.88	7.71	7.42
M-GalNAc(6S)_PCM_	0.82	3.13	0	4.04	4.39	5.44	9.48	5.42	3.09
GalNAc(46S)	0	6.15	6.43	5.60	29.94	25.15	3.48	19.74	3.13
M-GalNAc(46S)	3.14	8.75	0	5.11	22.36	13.03	4.59	12.43	15.29
GalNAc(46S)_PCM_	1.50	2.09	0	6.94	9.19	16.43	4.55	13.68	4.90
M-GalNAc(46S)_PCM_	0	2.35	2.74	5.13	6.53	10.39	11.72	6.32	2.99

Relative energies were calculated using the energy of the most stable conformation for the same molecule as a reference for *in  vacuo* and PCM solvent model (marked with PCM subscript).

^4^C_1_: The ring conformation changed to ^4^C_1_ during geometry optimization.

**Table 2:** B3LYP6311ppdd//B3LYP/6-31+G(d) relative energies for ring conformations of IdoUA and GlcUA derivatives.

Molecule/conformation	Δ*E* (^4^C_1_), kcal/mol	Δ*E* (^1^C_4_), kcal/mol	Δ*E* (^2^S_0_), kcal/mol
GlcUA	0.88	0	3.42
M-GlcUA	0	0.70	5.12
GlcUA_PCM_	0	1.69	3.11
M-GlcUA_PCM_	0	6.23	6.93
GlcUA(2S)	1.11	4.00	0
M-GlcU(2S)	0	5.29	6.84
GlcUA(2S)_PCM_	1.25	7.51	0
M-GlcU(2S)_PCM_	0	3.71	7.84
GlcU(3S)	4.44	0	1.93
M-GlcU(3S)	0	2.34	0.73
GlcU(3S)_PCM_	6.78	3.65	0
M-GlcU(3S)_PCM_	0	11.09	1.29
GlcUA(23S)	7.94	13.14	0
M-GlcUA(23S)	0	14.86	5.83
GlcUA(23S)_PCM_	5.82	10.89	0
M-GlcUA(23S)_PCM_	0	8.87	1.62
IdoUA	0	2.00	6.53
M-IdoUA	0	2.33	6.64
IdoUA_PCM_	0	1.54	5.59
M-IdoUA_PCM_	0	1.54	4.01
IdoUA(2S)	0.28	0	5.78
M-IdoUA(2S)	2.77	0	7.18
IdoUA(2S)_PCM_	0	4.71	7.18
M-IdoUA(2S)_PCM_	1.50	0	3.50
IdoUA(3S)	0	19.64	22.68
M-IdoUA(3S)	0	21.36	24.17
IdoUA(3S)_PCM_	0	1.99	2.94
M-IdoUA(3S)_PCM_	0	1.87	2.94
IdoUA(23S)	0	0.70	0.46
M-IdoUA(23S)	0	11.18	6.44
IdoUA(23S)_PCM_	0	5.55	7.57
M-IdoUA(23S)_PCM_	0	7.82	4.38

Relative energies were calculated using the energy of the most stable conformation for the same molecule as a reference for *in  vacuo* and PCM solvent model (marked with PCM subscript).

**Table 3:** *ω* dihedral angle *gg/gt/tg* (%) conformations distribution for GlcNAc/GalNAc monosaccharide derivatives.

Monosaccharide	*gg *	*gt *	*tg *
GlcNAc	50	48	2
GlcNAc(4S)	38	59	3
GlcNAc(6S)	78	12	11
GlcNAc(46S)	59	29	12
GalNAc	7	76	16
GalNAc(4S)	5	81	14
GalNAc(6S)	3	19	78
GalNAc(46S)	1	31	68

**Table 4:** *ω* dihedral angle *gg/gt/tg* (%) conformations distribution for GlcNAc/GalNAc monosaccharide units within hexameric GAGs.

^ 1^GAG	*gg *	*gt *	*tg *
HA	53	45	2
HA4	61	37	2
HA6	87	11	2
HA46	57	40	4
HA462′	83	16	1
HA463′	59	30	11
HA462′3′	34	54	12

CS_de	8	80	12
CS4	6	86	9
CS6	6	56	38
CS46	2	77	21
CS462′	0	81	19
CS463′	2	53	45
CS462′3′	2	57	42

HA, HA4, HA6, HA46, HA462′, HA463′, HA462′3′, CS, CS4, CS6, CS46, CS462′, CS463′, CS462′3′ stay for (GlcUA-GlcNAc)_3_, (GlcUA-GlcNAc(4S))_3_, (GlcUA-GlcNAc(6S))_3_, (GlcUA-GlcNAc(46S))_3_, (GlcUA(2S)-GlcNAc(46S))_3_, (GlcUA(3S)-GlcNAc(46S))_3_, (GlcUA(23S)-GlcNAc(46S))_3_, (GlcUA-GalNAc)_3_, (GlcUA-GalNAc(4S))_3_, (GlcUA-GalNAc(6S))_3_, (GlcUA-GalNAc(46S))_3_, (GlcUA(2S)-GalNAc(46S))_3_, (GlcUA(3S)-GalNAc(46S))_3_, and (GlcUA(23S)-GalNAc(46S))_3_, respectively.

**Table 5:** Experimentally versus computationally obtained chemical shifts (ppm).

Atom	GlcNAc		GlcNAc(6S)		GalNAc		GalNAc(4S)		GalNAc(6S)	
	Exp.	GIAO	Exp.	GIAO	Exp.	GIAO	Exp.	GIAO	Exp.	GIAO
C1	97.77	107.00	97.90	104.27	98.27	107.12	98.16	101.40	98.28	107.79
C2	59.60	64.30	59.58	65.23	56.62	62.65	56.98	60.46	56.45	62.80
C3	76.74	82.51	76.61	82.45	73.99	77.19	72.81	80.51	73.79	77.32
C4	72.76	80.69	72.58	78.70	70.77	74.61	78.64	77.33	70.48	72.60
C5	78.79	84.15	76.69	87.11	78.03	78.54	77.22	81.88	75.63	79.38
C6	63.64	69.17	70.13	71.15	64.06	68.44	63.85	65.28	70.30	70.57
C7	177.59	183.04	—	184.56	—	182.75	177.53	182.10	—	182.71
C8	25.03	23.96	24.93	24.01	24.97	24.08	25.08	24.96	25.08	24.07
H1	4.72	4.65	4.74	4.88	4.65	4.45	4.72	4.94	4.67	4.46
H2	3.67	3.78	3.70	3.67	3.88	3.94	3.89	4.00	3.89	3.90
H3	3.54	3.36	3.56	3.41	3.73	3.51	3.88	3.88	3.75	3.42
H4	3.46	3.17	3.52	3.27	3.94	4.35	4.70	5.21	4.00	4.24
H5	3.47	3.29	3.68	3.82	3.70	3.91	3.82	3.95	3.94	3.64
H6	3.75	3.84	4.22	4.25	3.77	4.15	—	3.84	4.20	4.12
H7	3.91	3.99	4.35	4.69	3.80	4.33	—	4.27	4.23	4.77
H9, 10, 11	2.05	2.05	2.05	2.12	2.06	2.05	2.06	2.11	2.06	2.02

**Table 6:** Experimentally versus computationally obtained ^3^J_H-H_ (Hz).

Protonpair	GlcNAc		GlcNAc(6S)		GalNAc		GalNAc(4S)		GalNAc(6S)	
	Exp.	GIAO	Exp.	GIAO	Exp.	GIAO	Exp.	GIAO	Exp.	GIAO
H1-H2	8.42	5.59	8.32	5.74	8.17	5.53	8.30	5.27	8.09	5.63
H2-H3	10.46	7.68	10.39	7.42	10.92	7.87	—	8.50	10.86	7.69
H3-H4	8.82	5.89	8.99	6.07	3.36	3.90	2.50	3.71	3.50	3.73
H4-H5	9.53	6.69	10.02	7.41	1.03	2.08	—	2.85	0.85	1.94
H5-H6	2.40	2.56	1.84	0.54	4.41	5.65	—	6.84	4.77	4.14
H5-H7	5.67	6.70	5.59	4.60	7.79	6.40	—	4.90	7.55	7.89

**Table 7:** Comparison of the experimental and theoretical data on NMR parameters.

	GlcNAc	GlcNAc(6S)	GalNAc	GalNAc(4S)	GalNAc(6S)	All
*R*_Pearson_, ^13^C	0.999 (0.998)	0.994	0.995	0.993 (0.998)	0.995	0.994 (0.996)
*R*_Spearman_, ^13^C	1.000	1.000	1.000	0.893 (0.929)	1.000	0.959 (0.965)
*R*_Pearson_, ^1^H	0.980	0.980	0.932	0.991	0.939	0.961
*R*_Spearman_, ^1^H	1.000	0.976	0.905	0.886	0.929	0.933
ΔΔppm, ^13^C	5.66 (5.63)	5.19	3.96	3.13 (4.39)	3.79	4.34 (4.38)
ΔΔppm, ^1^H	0.06	0.04	0.15	0.17	0.02	0.05
*R*_Pearson_, ^3^J_H-H_	0.836	0.985	0.933	—	0.923	0.899
*R*_Spearman_, ^3^J_H-H_	0.657	1.000	0.829	—	0.829	0.840
ΔΔppm, ^3^J_H-H_	1.7	2.2	0.7	0.9	0.8	1.3

The analysis for ^13^C chemical shifts is done without the consideration of C7 chemical shifts. The values obtained with the consideration of C7 chemical shifts from GlcNAc and GalNAc(4S) are given in the parenthesis.
