# Supplementary material for: Glycosaminoglycan Monosaccharide Blocks Analysis by Quantum Mechanics, Molecular Dynamics, and Nuclear Magnetic Resonance
Source: Biomed Res Int. 2014 Apr 7;2014:808071. doi: 10.1155/2014/808071 (PMC3997902; doi:10.1155/2014/808071)
Supplement: Supplementary file 1 — Supplementary Tables 1–12: The description should be "Chemical shifts calculated by GIAO approach". Supplementary Tables 13–17: The description should be "Chemical shifts calculated by GIAO approach". Supplementary Tables 13–16: The description should be "3JH-H calculated by GIAO approach". Supplementary Table 18: The description should be "3JH-H calculated by GIAO approach". Supplementary Table 19: 1H chemical shifts in CH3 of the acetyl group of Glc/GalNAc derivatives for gg/gt/tg conformations. Supplementary Table 18: 1H chemical shifts in CH3 of the acetyl group of Glc/GalNAc derivatives. Supplementary Table 19: 1H chemical shifts in CH3 of the acetyl group of Glc/GalNAc derivatives for gg/gt/tg conformations. [file 808071.f1.pdf]

Supplementary Materials

Supplementary Table 1. Chemical shifts (ppm) calculated by GIAO approach (B3LYP/6-311++G(2d,p)) for GlcNAc

Atom	GlcNAc									M-GlcNAc								
	$^{4}C_{1}$			$^{1}C_{4}$			$^{2}S_{0}$			$^{4}C_{1}$			$^{1}C_{4}$			$^{2}S_{0}$		
	gg	gt	tg	gg	gt	tg	gg	gt	tg	gg	gt	tg	gg	gt	tg	gg	gt	tg
C1	107.06	107.00	107.14	100.30	102.33	101.82	103.96	105.09	108.58	108.49	108.40	111.77	106.59	108.98	108.34	109.38	111.36	113.20
C2	64.52	64.30	63.63	51.89	52.38	53.69	68.81	67.77	71.97	55.66	55.75	57.56	53.06	54.61	54.19	69.72	69.12	64.04
C3	82.59	82.51	80.38	73.95	75.39	76.14	85.07	85.29	76.26	85.28	84.97	82.35	72.08	73.02	75.93	86.48	85.30	81.18
C4	76.93	80.69	84.26	82.57	77.04	76.34	69.73	72.18	79.78	76.64	80.61	84.01	80.11	78.06	76.24	69.37	72.53	77.33
C5	84.15	84.15	77.33	87.19	84.04	84.54	84.54	86.93	85.21	84.73	85.22	77.14	88.75	89.89	84.68	86.20	86.47	83.61
C6	65.26	69.17	74.21	70.45	68.42	70.14	65.85	72.80	74.33	66.22	69.16	73.99	69.23	71.06	69.29	68.51	71.39	74.52
C7	183.11	183.04	182.75	173.81	174.05	173.90	178.93	179.09	180.38	175.34	175.64	174.58	174.90	174.94	174.21	178.60	178.94	176.43
C8	23.92	23.96	23.99	24.29	24.29	24.44	24.20	24.14	24.03	24.68	24.64	24.60	24.42	24.20	24.50	24.10	24.23	24.61
H1	4.73	4.65	4.67	5.52	5.46	5.23	5.07	5.12	5.04	4.41	4.48	4.17	4.76	5.00	4.65	4.46	4.45	4.28
H2	3.76	3.78	3.74	4.55	4.64	4.64	3.82	3.92	3.75	4.58	4.53	4.48	4.27	4.47	4.51	3.90	3.88	4.16
H3	3.37	3.36	3.69	4.02	4.11	4.27	3.67	3.68	3.89	3.18	3.21	3.50	3.92	4.05	4.21	3.66	3.68	3.71
H4	3.95	3.17	3.74	4.30	4.03	4.43	4.92	4.40	4.28	4.16	3.19	3.80	4.20	4.08	4.37	4.71	4.18	4.43
H5	3.03	3.29	3.64	3.89	3.87	3.85	3.84	3.94	4.04	3.15	3.34	3.62	3.67	3.94	3.82	3.60	3.90	3.90
H6	3.97	3.84	4.09	4.20	4.27	4.00	3.92	4.14	4.21	4.38	3.88	4.10	4.03	4.30	4.10	3.78	4.09	4.17
H7	4.08	3.99	4.41	3.93	3.91	5.29	4.31	3.76	4.44	4.20	4.07	4.39	3.54	3.45	5.22	4.29	3.78	4.36
H8	5.74	5.67	5.78	6.74	6.83	6.89	5.41	5.35	5.14	4.63	4.64	4.51	6.84	7.15	6.91	5.47	5.41	5.14
H9	1.70	1.69	1.70	1.52	1.53	1.54	1.62	1.61	1.65	1.53	1.54	1.49	1.54	1.56	1.55	1.60	1.60	1.69
H10	2.21	2.22	2.21	2.07	2.07	2.05	2.19	2.23	2.16	2.19	2.20	2.08	2.06	2.06	2.06	2.22	2.23	2.19
H11	2.25	2.23	2.21	2.09	2.12	2.09	2.22	2.24	2.26	2.24	2.24	2.20	2.13	2.11	2.08	2.23	2.24	2.21

Supplementary Table 2. Chemical shifts (ppm) calculated by GIAO approach (B3LYP/6-311++G(2d,p)) for GlcNAc(4S)

Atom	GlcNAc(4S)									M-GlcNAc(4S)								
	$^4C_1$			$^1C_4$			$^2S_0$			$^4C_1$			$^1C_4$			$^2S_0$		
	gg	gt	tg	gg	gt	tg	gg	gt	tg	gg	gt	tg	gg	gt	tg	gg	gt	tg
C1	106.48	106.52	101.81	98.21	102.31	101.89	104.43	103.31	106.87	108.16	111.30	104.75	105.00	108.29	107.68	110.08	111.20	110.09
C2	64.86	65.05	66.90	54.02	52.69	53.84	65.21	66.00	71.67	56.29	59.26	60.59	56.78	53.84	54.32	66.28	65.83	65.18
C3	75.25	75.22	81.84	70.12	75.79	73.08	74.82	82.57	73.50	77.50	77.47	83.88	69.25	73.86	73.01	77.34	80.09	79.44
C4	88.38	92.47	85.42	88.13	80.02	80.22	88.93	83.25	88.71	88.46	92.14	86.28	85.89	80.67	80.12	75.63	82.91	85.18
C5	78.47	78.72	84.13	81.90	77.77	83.15	89.70	82.54	85.97	78.69	78.78	84.60	81.91	83.31	83.44	86.44	85.14	83.57
C6	65.48	69.09	70.98	71.27	67.51	71.71	69.67	72.64	79.38	66.56	69.66	70.74	72.17	71.12	70.96	69.46	71.30	78.19
C7	182.51	182.52	182.65	176.71	173.85	174.69	181.61	175.76	181.81	174.48	174.31	182.12	176.55	174.40	174.77	177.74	176.97	177.28
C8	23.97	24.03	24.91	24.07	24.37	24.25	24.17	24.32	23.84	24.72	24.62	24.76	24.11	24.34	24.27	24.21	24.29	24.19
H1	4.83	4.77	4.78	6.01	5.47	5.43	5.25	5.46	5.16	4.41	4.27	4.59	5.31	4.94	4.85	4.71	4.73	4.67
H2	3.63	3.67	3.45	4.19	4.51	4.54	3.61	4.30	3.39	4.54	4.46	3.74	4.07	4.38	4.42	4.00	4.05	4.02
H3	4.74	4.74	3.51	4.84	4.08	4.65	4.12	3.71	3.98	4.44	4.44	3.85	4.72	4.09	4.61	3.68	3.81	3.70
H4	4.43	3.66	4.23	4.80	4.67	5.11	5.08	5.05	5.04	4.55	3.68	4.74	4.80	4.64	5.12	5.06	4.75	5.35
H5	4.17	4.35	3.82	4.09	4.95	4.15	3.90	3.75	3.80	4.26	4.36	3.58	4.02	4.74	4.11	4.01	3.79	3.51
H6	4.05	3.69	4.06	4.18	4.00	4.66	4.05	3.81	4.33	4.46	3.76	4.21	4.21	3.94	4.62	3.88	3.59	4.31
H7	4.08	4.13	3.93	3.92	4.67	4.02	3.51	3.91	4.32	4.17	4.20	3.90	3.92	4.61	4.09	4.74	4.22	4.18
H8	5.86	5.77	5.74	6.86	6.52	7.08	6.55	5.10	6.07	4.81	4.48	5.91	6.98	6.83	7.12	5.34	5.22	5.18
H9	1.68	1.68	1.76	1.81	1.58	1.70	1.74	1.57	1.72	1.56	1.48	1.75	1.80	1.62	1.71	1.66	1.61	1.60
H10	2.16	2.19	2.24	1.91	2.03	1.09	2.16	2.06	2.16	2.19	2.11	2.24	1.93	2.01	1.99	2.13	2.10	2.08
H11	2.21	2.21	2.30	2.02	2.11	2.09	2.18	2.19	2.19	2.19	2.17	2.26	1.97	2.11	2.08	2.23	2.21	2.18

Supplementary Table 3. Chemical shifts (ppm) calculated by GIAO approach (B3LYP/6-311++G(2d,p)) for GlcNAc(6S)

Atom	GlcNAc(6S)									M-GlcNAc(6S)								
	$^{4}C_{1}$			$^{1}C_{4}$			$^{2}S_{0}$			$^{4}C_{1}$			$^{1}C_{4}$			$^{2}S_{0}$		
	gg	gt	tg	gg	gt	tg	gg	gt	tg	gg	gt	tg	gg	gt	tg	gg	gt	tg
C1	104.16	104.27	107.93	100.62	103.05	102.83	107.55	106.24	106.85	109.72	111.25	104.06	105.74	108.67	109.08	108.42	110.57	112.61
C2	65.71	65.23	62.81	50.60	51.82	54.19	64.34	68.14	68.84	56.49	61.90	60.08	52.81	54.00	54.58	61.00	69.54	64.15
C3	81.49	82.45	81.85	76.90	74.49	75.05	81.98	84.24	73.86	84.77	83.53	81.62	76.66	73.59	74.94	85.35	85.48	80.72
C4	74.37	78.70	85.70	77.92	78.75	73.73	80.89	74.04	81.38	75.82	79.15	78.34	77.19	79.34	73.55	78.88	71.93	77.01
C5	86.78	87.11	76.13	88.57	87.56	82.72	85.41	82.44	89.45	85.46	89.07	80.00	87.86	86.12	82.86	78.82	85.90	85.90
C6	69.53	71.15	73.52	71.92	79.50	74.31	74.90	79.47	80.30	68.23	71.46	73.87	72.32	75.70	73.73	69.96	78.13	79.46
C7	184.44	184.56	182.66	174.26	173.41	173.99	179.50	178.75	180.56	177.29	178.48	180.86	174.58	174.33	173.90	173.57	178.50	176.19
C8	24.54	24.01	23.94	24.30	24.37	24.51	24.00	24.26	24.08	24.57	24.41	24.60	24.35	24.31	24.58	24.45	24.13	24.65
H1	4.99	4.88	4.63	5.54	5.51	5.25	5.29	5.12	5.22	4.78	4.27	4.55	4.99	4.98	4.61	4.86	4.40	4.21
H2	3.71	3.67	3.79	4.74	4.56	4.54	4.06	3.71	3.31	4.70	4.30	3.68	4.54	4.38	4.43	4.47	3.82	4.15
H3	3.34	3.41	3.65	4.11	4.07	4.38	3.46	3.73	3.86	3.16	3.31	3.84	4.02	4.06	4.32	3.64	3.63	3.68
H4	4.60	3.27	3.88	5.05	4.10	4.86	4.32	4.26	4.15	4.02	3.25	4.17	5.20	4.21	4.82	4.66	4.22	4.39
H5	3.24	3.82	3.50	3.97	3.97	3.78	4.24	3.92	4.11	3.10	3.69	3.83	3.93	4.01	3.75	3.72	3.97	3.85
H6	4.22	4.69	4.91	4.10	3.92	5.94	4.57	4.90	4.22	4.43	4.76	5.08	4.12	4.15	5.88	4.32	4.84	4.09
H7	4.96	4.25	4.40	4.51	5.37	4.03	4.42	4.38	5.09	5.21	4.30	4.52	4.47	4.64	4.07	4.48	4.42	5.09
H8	6.24	6.04	5.66	6.83	7.02	6.89	6.05	5.33	6.14	4.60	4.83	5.94	7.03	7.21	6.93	5.47	5.44	5.11
H9	1.77	1.76	1.67	1.56	1.51	1.52	1.66	1.59	1.68	1.64	1.72	1.72	1.58	1.57	1.54	1.50	1.59	1.60
H10	2.22	2.27	2.21	2.11	2.05	2.00	2.19	2.17	2.15	2.40	2.11	2.25	2.10	2.05	2.02	2.13	2.17	2.18
H11	2.32	2.32	2.26	2.14	2.05	2.07	2.19	2.20	2.19	2.15	2.37	2.28	2.12	2.09	2.07	2.17	2.19	2.19

Supplementary Table 4. Chemical shifts (ppm) calculated by GIAO approach (B3LYP/6-311++G(2d,p)) for GlcNAc(46S)

Atom	GlcNAc(46S)									M-GlcNAc(46S)								
	$^{4}C_{1}$			$^{1}C_{4}$			$^{2}S_{0}$			$^{4}C_{1}$			$^{1}C_{4}$			$^{2}S_{0}$		
	gg	gt	tg	gg	gt	tg	gg	gt	tg	gg	gt	tg	gg	gt	tg	gg	gt	tg
C1	104.76	105.53	106.98	100.23	102.43	102.41	105.02	106.70	106.72	111.01	109.46	109.34	105.35	108.51	108.82	106.69	112.78	110.28
C2	67.15	64.62	63.00	52.55	55.50	59.90	67.56	58.74	62.20	58.39	57.49	56.19	53.28	56.90	60.35	63.13	58.50	67.86
C3	77.22	75.49	80.23	75.59	68.40	76.03	82.73	75.74	75.09	76.70	76.83	82.29	74.56	67.93	75.76	77.64	75.42	76.99
C4	78.01	86.00	91.32	84.92	77.75	79.01	85.17	85.54	91.21	85.04	86.16	91.71	81.04	78.53	78.47	86.78	85.38	89.85
C5	83.98	77.51	76.83	81.27	84.99	81.04	83.52	84.81	89.77	82.60	78.64	77.47	84.63	84.30	80.88	85.45	84.87	79.78
C6	72.68	74.48	75.84	77.65	79.46	69.87	79.60	79.39	77.02	71.20	75.15	75.00	72.68	74.81	69.15	79.48	79.56	75.70
C7	184.50	184.81	183.89	173.86	185.93	181.24	179.85	175.44	183.43	178.84	178.44	175.73	174.70	186.21	181.22	177.73	175.17	178.22
C8	24.52	24.06	24.03	24.52	26.89	24.46	24.04	24.55	24.29	24.27	24.55	24.53	24.35	27.09	24.43	25.05	24.58	24.49
H1	5.15	4.97	4.63	5.52	5.52	5.74	5.32	5.02	5.25	4.71	4.68	4.45	4.98	4.90	5.12	5.17	4.44	5.16
H2	3.69	3.60	3.73	4.47	3.91	4.19	4.45	4.49	3.31	4.19	4.28	4.55	4.46	3.93	4.06	4.33	4.47	3.56
H3	3.66	5.14	3.86	3.86	5.21	4.22	3.72	3.74	3.85	4.53	4.90	3.63	4.53	5.26	4.19	5.54	3.74	3.99
H4	5.42	3.58	4.56	5.02	4.39	5.61	5.30	4.69	4.79	4.15	3.54	4.71	5.59	4.48	5.58	6.42	4.66	4.72
H5	3.71	5.56	3.43	4.89	4.40	4.57	4.91	5.54	5.92	4.53	5.48	3.31	4.21	4.44	4.57	4.44	5.48	4.64
H6	4.92	4.90	4.81	4.24	4.12	5.63	5.28	4.93	4.61	5.13	5.09	4.79	4.14	4.20	5.51	4.34	4.77	4.76
H7	4.59	3.73	4.50	4.37	5.20	4.04	5.02	5.20	4.18	4.31	3.81	4.47	4.61	4.79	4.07	5.17	5.19	4.59
H8	6.38	6.44	5.88	6.39	5.53	5.31	5.47	4.44	6.58	5.07	4.90	4.67	6.74	5.58	5.28	5.51	4.45	5.77
H9	1.81	1.82	1.73	1.61	1.77	1.78	1.67	1.60	1.81	1.69	1.73	1.55	1.63	1.78	1.78	1.64	1.53	1.69
H10	2.25	2.21	2.20	2.03	1.76	2.14	2.24	2.22	2.26	2.15	2.12	2.16	2.06	1.82	2.13	2.12	2.20	2.09
H11	2.33	2.29	2.22	2.06	3.14	2.41	2.24	2.26	2.33	2.26	2.36	2.26	2.07	3.17	2.39	2.13	2.23	2.13

Supplementary Table 5. Chemical shifts (ppm) calculated by GIAO approach (B3LYP/6-311++G(2d,p)) for GalNAc

Atom	GalNAc									M-GalNAc								
	$^4C_1$			$^1C_4$			$^2S_0$			$^4C_1$			$^1C_4$			$^2S_0$		
	gg	gt	tg	gg	gt	tg	gg	gt	tg	gg	gt	tg	gg	gt	tg	gg	gt	tg
C1	107.61	107.80	107.12	100.53	102.48	104.36	104.64	106.10	107.42	109.99	110.06	108.17	107.27	109.16	110.27	112.74	112.45	111.88
C2	62.41	64.23	62.65	54.64	54.15	55.07	63.75	62.29	64.02	59.30	66.62	54.42	56.55	55.54	55.65	62.78	60.53	60.72
C3	78.92	78.73	77.19	72.65	76.81	79.42	72.89	72.15	75.23	84.62	75.70	79.41	73.41	75.82	79.59	71.49	82.17	81.74
C4	78.46	76.07	74.61	69.47	70.96	74.89	76.88	75.82	74.13	81.12	77.96	74.87	70.43	71.08	75.34	75.58	72.82	72.77
C5	78.45	82.35	78.54	83.86	78.42	79.00	83.40	84.42	83.35	81.14	85.08	79.33	83.89	84.94	78.83	87.38	87.08	82.78
C6	71.58	67.57	68.44	65.97	68.14	73.41	69.71	66.99	68.83	72.12	70.78	67.70	67.16	68.84	72.64	66.45	69.41	68.67
C7	183.50	183.47	182.75	173.92	173.75	173.12	182.02	181.03	182.28	180.58	176.29	175.65	173.09	173.58	173.41	177.40	179.98	179.97
C8	24.11	24.01	24.08	24.31	24.32	24.51	24.01	24.09	24.26	24.07	26.14	24.75	24.43	24.26	24.64	25.98	24.24	24.22
H1	4.63	4.58	4.45	5.25	5.34	5.15	5.21	5.20	5.08	4.48	4.23	4.35	4.61	4.75	4.51	4.55	4.39	4.27
H2	4.04	3.60	3.94	4.76	4.89	5.10	3.47	3.66	4.26	4.65	3.13	4.70	4.75	4.79	5.04	4.11	4.08	3.98
H3	3.33	3.36	3.51	4.03	3.99	4.08	3.60	3.76	3.87	3.38	4.01	3.29	3.79	3.95	4.04	4.42	3.79	3.83
H4	4.31	3.77	4.35	4.16	4.03	3.88	4.56	4.67	4.81	4.35	3.93	4.40	4.19	4.04	3.85	4.83	4.34	4.54
H5	3.41	3.65	3.91	4.32	4.53	4.46	4.32	4.39	4.50	3.31	3.69	3.90	4.13	4.53	4.44	4.29	4.13	4.28
H6	4.05	4.10	4.15	3.88	4.46	4.20	4.14	4.38	3.76	4.06	4.28	4.15	4.03	4.38	4.30	3.83	4.50	3.73
H7	4.34	3.66	4.33	4.67	4.20	5.31	4.10	3.67	4.81	4.36	4.11	4.28	4.45	4.37	5.27	4.20	3.69	4.57
H8	5.67	5.61	5.67	5.16	5.21	5.17	6.15	6.16	4.95	5.03	5.71	4.59	5.17	5.23	5.20	5.51	5.19	5.22
H9	1.74	1.73	1.72	1.51	1.51	1.46	1.76	1.73	1.68	1.60	1.57	1.55	1.49	1.51	1.47	1.57	1.65	1.62
H10	2.23	2.23	2.18	2.14	2.14	2.12	2.19	2.18	2.25	2.17	2.20	2.18	2.13	2.11	2.11	2.21	2.26	2.23
H11	2.24	2.26	2.24	2.16	2.16	2.21	2.23	2.23	2.31	2.20	2.21	2.23	2.16	2.16	2.21	2.21	2.26	2.26

Supplementary Table 6. Chemical shifts (ppm) calculated by GIAO approach (B3LYP/6-311++G(2d,p)) for GalNAc(4S)

Atom	GalNAc(4S)									M-GalNAc(4S)								
	$^{4}C_{1}$			$^{1}C_{4}$			$^{2}S_{0}$			$^{4}C_{1}$			$^{1}C_{4}$			$^{2}S_{0}$		
	gg	gt	tg	gg	gt	tg	gg	gt	tg	gg	gt	tg	gg	gt	tg	gg	gt	tg
C1	105.90	108.34	101.40	99.78	103.20	100.34	105.84	110.95	109.12	113.78	107.68	104.41	107.57	109.73	107.09	115.45	112.43	114.76
C2	67.50	62.66	60.46	63.22	57.31	59.54	63.18	62.13	60.87	58.12	58.97	54.89	57.02	57.94	59.37	65.03	63.96	60.99
C3	78.37	78.49	80.51	75.25	74.58	73.83	72.47	76.88	75.22	79.91	82.05	81.52	74.97	73.68	73.81	76.76	76.15	75.46
C4	79.72	82.55	77.33	78.15	82.68	76.58	80.68	84.12	79.98	80.69	78.23	77.24	72.48	82.50	76.55	80.91	83.49	80.22
C5	80.39	81.13	81.88	81.71	76.85	81.25	85.99	90.95	87.65	81.38	84.02	82.04	82.21	80.98	81.46	86.80	86.17	87.08
C6	68.57	68.39	65.28	64.38	68.26	76.92	68.20	72.74	67.43	68.61	69.03	65.23	69.59	68.97	76.38	66.28	69.87	66.76
C7	183.94	183.62	182.10	174.80	173.33	175.07	183.20	183.48	183.38	176.44	181.74	182.04	174.90	173.00	174.78	180.01	177.60	183.00
C8	24.31	24.09	24.96	24.20	24.44	24.39	23.96	24.49	24.39	24.46	25.18	25.08	24.15	24.44	24.30	24.41	24.52	24.49
H1	4.95	4.59	4.94	4.89	5.28	5.50	5.23	5.44	5.23	4.13	4.43	4.67	4.61	4.65	4.86	5.22	5.27	4.57
H2	3.53	4.03	4.00	4.16	4.79	4.53	3.69	4.47	4.50	4.48	4.03	4.26	4.53	4.78	4.41	4.15	3.99	4.43
H3	3.35	3.59	3.88	4.51	4.69	4.74	3.89	3.85	3.92	3.22	3.80	3.86	5.41	4.72	4.71	3.53	3.66	3.96
H4	4.86	4.78	5.21	5.17	4.80	4.60	5.47	5.05	5.24	4.89	4.95	5.20	4.89	4.77	4.58	4.86	5.19	5.27
H5	3.25	3.77	3.95	4.50	4.41	3.87	4.53	4.39	4.35	3.20	3.79	3.95	4.26	4.32	3.84	4.39	4.26	4.32
H6	4.25	3.69	4.27	3.69	5.55	5.62	4.08	4.13	4.15	4.31	3.82	4.24	3.96	5.29	5.59	3.88	3.49	4.13
H7	4.01	4.05	3.84	4.35	3.91	4.18	3.85	4.25	3.75	4.01	4.48	3.90	4.09	4.00	4.09	3.98	4.40	3.74
H8	5.66	5.97	5.38	5.34	5.21	5.08	6.51	6.11	5.88	4.53	5.17	5.43	5.30	5.29	5.07	5.84	5.78	5.91
H9	1.77	1.76	1.70	1.57	1.52	1.56	1.84	1.80	1.74	1.55	1.64	1.73	1.57	1.52	1.54	1.79	1.69	1.72
H10	2.22	2.18	2.31	2.11	2.04	2.07	2.19	2.22	2.27	2.14	2.28	2.28	2.10	2.05	2.08	2.14	2.06	2.24
H11	2.32	2.20	2.31	2.17	2.19	2.15	2.25	2.36	2.40	2.29	2.29	2.36	2.32	2.21	2.14	2.25	2.11	2.41

Supplementary Table 7. Chemical shifts (ppm) calculated by GIAO approach (B3LYP/6-311++G(2d,p)) for GalNAc(6S)

Atom	GalNAc(6S)									M-GalNAc(6S)								
	$^{4}C_{1}$			$^{1}C_{4}$			$^{2}S_{0}$			$^{4}C_{1}$			$^{1}C_{4}$			$^{2}S_{0}$		
	gg	gt	tg	gg	gt	tg	gg	gt	tg	gg	gt	tg	gg	gt	tg	gg	gt	tg
C1	104.71	104.63	107.79	100.34	103.03	103.26	104.28	104.87	106.38	110.07	108.22	108.83	108.32	109.85	109.83	109.15	110.01	110.31
C2	63.50	64.97	62.80	58.38	54.89	58.23	66.06	60.86	62.91	53.94	55.31	54.87	57.16	55.24	58.85	57.17	58.69	63.26
C3	78.89	78.58	77.32	75.48	75.27	78.10	74.69	80.49	72.79	81.96	81.26	79.42	75.26	76.21	78.11	79.13	81.41	76.43
C4	76.67	77.82	72.60	72.79	71.31	78.69	74.01	73.87	73.80	78.28	79.28	72.72	72.42	71.69	78.70	73.79	72.58	74.26
C5	80.80	86.17	79.38	82.51	83.91	81.82	80.45	80.78	83.79	80.58	86.80	79.94	84.50	83.78	82.09	82.51	82.64	81.69
C6	74.74	72.63	70.57	68.22	78.23	73.90	73.88	74.21	75.63	75.36	73.79	70.31	71.41	73.93	73.26	73.78	70.95	73.74
C7	184.52	185.13	182.71	174.47	173.47	172.94	180.40	181.76	180.47	177.87	178.28	175.47	172.63	173.29	172.90	173.95	181.65	176.79
C8	24.55	24.12	24.07	24.02	24.39	24.27	24.31	24.18	24.07	24.59	24.62	24.69	24.67	24.38	24.33	24.44	24.00	24.50
H1	4.90	4.78	4.46	5.34	5.39	5.11	5.27	5.12	5.12	4.77	4.60	4.32	4.59	4.67	4.50	4.40	4.64	4.99
H2	3.87	3.41	3.90	4.30	4.77	4.93	5.09	4.28	3.74	4.98	4.35	4.64	4.82	4.93	4.84	5.75	4.54	3.82
H3	3.28	3.38	3.42	4.08	3.98	4.53	3.68	3.94	3.70	3.09	3.30	3.23	3.81	3.85	4.48	3.61	3.92	3.53
H4	4.39	3.91	4.24	4.55	4.08	4.04	4.53	4.47	4.57	4.36	4.00	4.29	4.04	4.03	4.03	4.34	4.57	4.41
H5	3.47	4.20	3.64	4.29	4.43	3.73	4.10	4.60	4.40	3.40	4.51	3.65	4.16	4.42	3.70	4.08	4.83	4.15
H6	4.34	4.81	4.77	4.79	4.62	6.12	4.89	5.00	4.31	4.32	4.71	4.77	4.07	4.74	6.03	4.45	4.93	4.08
H7	5.41	4.03	4.12	4.33	5.19	4.11	4.93	4.38	5.65	5.42	4.17	4.08	4.96	4.49	4.14	5.11	4.40	5.64
H8	6.18	5.91	5.64	5.18	5.17	5.00	5.36	5.12	5.99	4.61	4.76	4.62	5.18	5.24	5.02	4.77	5.04	5.48
H9	1.82	1.81	1.71	1.53	1.51	1.45	1.67	1.65	1.70	1.63	1.65	1.55	1.49	1.49	1.45	1.46	1.60	1.64
H10	2.26	2.31	2.16	2.18	2.09	2.13	2.18	2.23	2.14	2.18	2.21	2.13	2.07	2.12	2.12	2.20	2.21	2.08
H11	2.32	2.32	2.20	2.18	2.15	2.15	2.30	2.26	2.18	2.38	2.38	2.23	2.19	2.18	2.15	2.22	2.33	2.08

Supplementary Table 8. Chemical shifts (ppm) calculated by GIAO approach (B3LYP/6-311++G(2d,p)) for GalNAc(46S)

Atom	GalNAc(46S)									M-GalNAc(46S)								
	$^4C_1$			$^1C_4$			$^2S_0$			$^4C_1$			$^1C_4$			$^2S_0$		
	gg	gt	tg	gg	gt	tg	gg	gt	tg	gg	gt	tg	gg	gt	tg	gg	gt	tg
C1	105.58	107.39	108.15	101.58	102.65	102.95	108.31	102.39	108.93	111.31	113.72	109.40	104.89	108.93	108.10	113.09	112.17	111.81
C2	63.70	60.96	61.91	53.63	54.81	57.25	61.40	67.50	67.68	55.87	55.12	54.44	63.54	54.96	58.11	66.85	63.96	62.38
C3	77.19	80.14	78.24	72.92	78.34	77.48	72.55	66.27	76.10	80.10	82.20	80.33	67.95	78.57	77.51	85.06	67.01	75.51
C4	77.83	80.31	83.84	74.80	74.34	84.57	79.51	75.88	83.26	78.69	79.98	84.17	76.97	74.69	84.70	84.65	80.01	79.02
C5	81.65	79.40	76.57	79.26	78.37	82.22	84.07	84.66	82.06	80.51	79.20	77.17	81.58	80.43	82.00	84.64	86.97	80.38
C6	73.48	67.34	72.53	69.61	74.66	72.26	68.65	74.81	70.83	74.12	67.50	72.13	69.63	72.86	71.46	68.63	77.31	74.68
C7	185.08	184.85	183.33	173.61	174.83	174.88	183.55	182.12	182.45	178.57	179.71	176.07	182.87	174.81	174.91	183.03	179.68	176.95
C8	24.42	24.36	23.94	24.43	24.27	23.98	24.95	25.32	23.71	24.57	24.37	24.80	26.69	24.19	24.03	24.63	25.09	24.83
H1	4.91	4.83	4.49	5.26	5.43	5.35	5.08	7.13	5.47	4.77	4.60	4.31	4.73	4.79	4.69	4.45	6.05	5.21
H2	3.97	4.72	4.02	5.08	4.84	4.76	4.20	2.93	4.30	4.76	5.22	4.77	4.04	4.87	4.65	4.38	2.90	3.95
H3	3.51	3.57	3.50	4.74	4.27	5.15	4.00	4.71	3.74	3.37	3.49	3.39	5.67	4.39	5.09	4.10	4.70	3.63
H4	5.03	4.68	5.21	4.97	5.28	4.69	5.25	5.52	4.85	4.94	4.66	5.17	5.13	5.12	4.63	5.28	5.75	5.44
H5	3.64	4.02	4.36	4.42	4.49	4.53	4.62	5.49	4.59	3.60	3.92	4.34	4.47	4.54	4.44	4.09	4.89	4.54
H6	4.42	4.70	4.05	4.27	4.51	4.80	4.27	4.02	4.59	4.53	4.57	4.00	4.28	4.61	4.78	4.60	4.70	3.86
H7	4.96	4.25	4.92	4.15	4.87	4.36	4.57	5.59	4.40	4.95	4.19	4.90	4.44	4.71	4.51	4.22	5.53	5.38
H8	6.53	6.72	5.96	5.30	5.29	5.19	5.98	5.28	6.04	4.61	5.16	4.71	5.37	5.29	5.21	5.39	5.54	6.04
H9	1.84	1.87	1.74	1.55	1.60	1.55	1.83	1.86	1.78	1.68	1.76	1.56	1.78	1.60	1.55	1.73	1.75	1.70
H10	2.21	2.20	2.19	2.15	2.06	2.16	2.29	2.01	2.22	2.11	2.10	2.17	2.26	2.06	2.16	2.20	2.02	2.01
H11	2.35	2.35	2.21	2.35	2.29	2.21	2.35	2.45	2.24	2.43	2.36	2.22	2.28	2.32	2.22	2.37	2.37	2.11

Supplementary Table 9. Chemical shifts (ppm) calculated by GIAO approach (B3LYP/6-311++G(2d,p)) for GlcUA and IdoUA

Atom	GlcUA			M-GlcUA			IdoUA			M-IdoUA		
	$^4C_1$	$^1C_4$	$^2S_0$	$^4C_1$	$^1C_4$	$^2S_0$	$^4C_1$	$^1C_4$	$^2S_0$	$^4C_1$	$^1C_4$	$^2S_0$
C1	104.49	105.19	105.93	107.26	113.74	118.95	101.26	106.04	106.86	107.20	111.78	112.41
C2	83.30	72.76	82.67	74.19	76.58	79.82	85.08	74.57	83.91	80.65	75.46	83.66
C3	85.19	75.16	79.30	85.69	75.14	81.85	82.85	76.30	83.73	83.34	76.58	83.90
C4	79.25	81.87	87.00	79.56	78.08	80.77	78.29	79.06	80.41	78.21	79.15	80.32
C5	80.44	86.73	93.05	80.54	83.96	87.25	79.21	72.32	78.49	78.92	72.23	78.23
C6	192.07	193.81	196.03	192.32	186.62	191.77	193.01	193.79	191.01	193.66	193.81	191.45
H1	4.60	5.56	4.86	4.66	5.12	4.81	5.31	5.47	5.36	5.07	4.92	4.44
H2	3.27	3.77	3.60	4.01	3.57	4.61	3.17	3.83	3.48	3.56	3.72	3.42
H3	3.65	4.27	3.94	3.66	4.50	3.86	3.34	4.37	5.02	3.33	4.33	3.72
H4	3.59	4.84	4.28	3.57	5.62	4.96	3.71	3.82	3.48	3.74	3.79	4.20
H5	3.91	4.39	4.56	3.91	4.29	4.35	4.61	5.07	3.70	4.61	4.84	4.46

Supplementary Table 10. Chemical shifts (ppm) calculated by GIAO approach (B3LYP/6-311++G(2d,p)) for GlcUA(2S) and IdoUA(2S)

Atom	GlcUA(2S)			M-GlcUA(2S)			IdoUA(2S)			M-IdoUA(2S)		
	$^4C_1$	$^1C_4$	$^2S_0$	$^4C_1$	$^1C_4$	$^2S_0$	$^4C_1$	$^1C_4$	$^2S_0$	$^4C_1$	$^1C_4$	$^2S_0$
C1	103.72	105.83	106.25	110.67	109.82	115.82	102.09	104.27	108.09	107.12	110.27	111.57
C2	86.42	75.70	88.53	84.00	80.24	82.42	84.67	77.25	86.77	85.10	74.83	86.21
C3	87.11	78.57	75.59	88.53	70.84	88.20	82.85	76.37	79.86	86.57	72.49	79.92
C4	79.62	77.39	85.30	79.03	78.80	82.33	79.70	76.31	79.66	78.23	78.43	79.64
C5	79.40	82.66	93.78	77.73	80.89	88.91	77.31	71.00	79.44	77.14	71.08	78.78
C6	191.00	185.73	196.46	191.88	194.08	189.20	192.78	193.83	190.27	192.73	193.27	190.63
H1	4.83	5.65	5.44	4.39	4.88	4.76	5.04	5.53	5.73	5.00	5.00	5.12
H2	4.59	4.57	4.32	4.84	4.56	6.08	4.32	4.29	4.49	4.65	4.52	4.36
H3	3.99	4.28	3.92	4.02	5.38	3.34	3.49	4.63	4.01	3.76	4.82	4.00
H4	3.75	5.16	4.14	3.74	4.44	4.95	3.84	4.15	4.31	3.79	4.16	4.28
H5	3.87	4.52	4.78	3.76	4.43	4.42	4.58	5.08	4.53	4.58	4.68	4.38

Supplementary Table 11. Chemical shifts (ppm) calculated by GIAO approach (B3LYP/6-311++G(2d,p)) for GlcUA(3S) and IdoUA(3S)

Atom	GlcUA(3S)			M-GlcUA(3S)			IdoUA(3S)			M-IdoUA(3S)		
	$^4C_1$	$^1C_4$	$^2S_0$	$^4C_1$	$^1C_4$	$^2S_0$	$^4C_1$	$^1C_4$	$^2S_0$	$^4C_1$	$^1C_4$	$^2S_0$
C1	103.49	105.28	104.96	110.68	110.36	106.24	103.87	106.25	106.05	110.28	112.13	111.77
C2	82.17	78.24	81.88	79.69	74.85	90.16	83.13	74.24	81.60	80.64	75.47	81.29
C3	87.51	83.41	86.79	89.75	82.05	85.48	86.36	79.59	94.06	87.27	79.82	94.10
C4	77.31	78.82	86.61	76.71	78.74	85.48	76.30	72.66	79.54	76.02	72.97	79.37
C5	82.85	87.03	89.66	81.98	84.54	89.76	81.97	73.21	78.56	81.88	72.77	78.34
C6	191.62	189.17	195.35	192.53	185.16	195.66	190.14	193.92	191.60	190.89	193.83	191.87
H1	4.99	5.39	4.98	4.48	5.13	5.12	6.25	5.45	5.31	5.82	4.92	4.78
H2	3.54	3.86	3.94	3.72	4.05	3.36	3.44	3.81	3.88	3.51	3.68	3.84
H3	4.43	4.52	4.44	4.46	4.68	4.58	4.43	5.05	4.91	4.36	5.02	4.91
H4	4.25	5.22	4.65	4.19	5.57	4.33	3.66	4.72	4.18	3.64	4.74	4.15
H5	4.15	4.47	4.75	4.06	4.28	4.78	4.78	4.96	4.74	4.69	4.82	4.52

Supplementary Table 12. Chemical shifts (ppm) calculated by GIAO approach (B3LYP/6-311++G(2d,p)) for GlcUA(23S) and IdoUA(23S)

Atom	GlcUA(23S)			M-GlcUA(23S)			IdoUA(23S)			M-IdoUA(23S)		
	$^{4}C_{1}$	$^{1}C_{4}$	$^{2}S_{0}$	$^{4}C_{1}$	$^{1}C_{4}$	$^{2}S_{0}$	$^{4}C_{1}$	$^{1}C_{4}$	$^{2}S_{0}$	$^{4}C_{1}$	$^{1}C_{4}$	$^{2}S_{0}$
C1	106.78	97.91	106.13	111.01	103.63	115.64	102.18	100.07	105.87	112.60	104.25	112.50
C2	84.17	86.55	84.78	85.23	82.00	78.13	82.63	81.15	82.10	83.06	77.75	80.38
C3	86.38	86.46	85.30	92.54	85.04	85.59	90.11	85.69	90.86	87.21	84.49	92.83
C4	83.87	75.96	87.79	78.06	74.16	85.13	82.20	75.22	83.26	77.51	73.83	82.71
C5	84.02	91.78	90.07	82.04	89.88	92.82	76.73	77.10	74.27	81.34	79.54	73.91
C6	187.08	188.90	194.69	191.39	183.77	178.05	193.37	195.60	190.97	191.41	195.75	192.10
H1	5.03	7.10	4.95	4.36	6.70	4.85	4.91	6.72	5.54	5.58	6.46	4.88
H2	4.39	4.67	4.96	4.71	4.94	6.06	4.35	4.45	4.31	4.53	4.45	4.98
H3	4.47	4.42	4.43	4.79	4.58	4.02	4.56	5.08	4.81	4.54	4.95	4.70
H4	3.91	5.00	4.80	4.19	5.46	5.04	4.24	5.81	4.54	3.78	5.97	4.73
H5	4.01	4.78	4.79	4.09	4.44	3.91	4.52	6.18	4.87	4.80	6.60	4.62

Supplementary Table 13. $^{3}J_{H\text{-}H}$ (Hz) calculated by GIAO approach (B3LYP/6-311++G(2d,p)) for GlcNAc and GlcNAc(4S)

Atomic pair	GlcNAc									M-GlcNAc								
	$^{4}C_{1}$			$^{1}C_{4}$			$^{2}S_{0}$			$^{4}C_{1}$			$^{1}C_{4}$			$^{2}S_{0}$		
	gg	gt	tg	gg	gt	tg	gg	gt	tg	gg	gt	tg	gg	gt	tg	gg	gt	tg
H1-H2	5.54	5.59	5.55	1.94	1.69	1.17	0.04	0.02	0.20	6.11	6.24	5.77	1.07	1.66	1.19	0.16	0.08	0.02
H2-H3	7.69	7.68	7.92	2.41	2.32	1.91	2.64	2.59	6.54	7.44	7.41	8.02	1.77	2.56	1.94	2.18	2.63	4.34
H2-H8	0.82	0.94	0.93	6.45	6.80	7.31	2.07	2.80	6.84	7.62	7.57	7.50	6.72	6.08	7.34	2.58	2.98	8.29
H3-H4	6.13	5.89	5.73	2.16	2.65	2.41	6.95	6.89	7.01	6.19	5.96	5.75	2.37	2.80	2.42	6.93	6.97	7.25
H4-H5	6.42	6.69	6.15	0.10	0.69	1.22	6.64	6.80	4.70	6.58	6.82	6.26	0.97	0.36	1.23	7.26	6.80	5.18
H5-H6	1.03	6.70	7.07	3.56	8.48	3.77	2.86	2.52	4.33	2.29	6.66	7.13	2.43	8.12	3.80	1.55	2.68	4.19
H5-H7	2.51	2.56	4.12	1.22	6.09	7.83	1.15	7.21	7.85	1.77	2.42	4.09	1.67	2.85	7.85	2.00	7.36	7.71
Atomic pair	GlcNAc(4S)									M-GlcNAc(4S)								
	$^{4}C_{1}$			$^{1}C_{4}$			$^{2}S_{0}$			$^{4}C_{1}$			$^{1}C_{4}$			$^{2}S_{0}$		
	gg	gt	tg	gg	gt	tg	gg	gt	tg	gg	gt	tg	gg	gt	tg	gg	gt	tg
H1-H2	5.59	5.67	5.15	1.81	1.62	1.62	3.18	0.57	1.39	6.25	5.95	5.98	1.17	1.51	1.67	0.10	0.03	0.01
H2-H3	8.18	8.13	8.25	2.45	2.29	2.48	8.69	2.52	7.79	7.88	8.03	6.22	2.19	2.49	2.54	5.46	4.42	4.68
H2-H8	0.83	0.99	4.44	1.77	6.63	6.32	0.17	2.90	0.19	7.98	8.48	0.13	2.24	6.36	6.26	2.32	2.77	2.22
H3-H4	7.22	6.93	7.21	2.42	3.21	3.27	5.69	6.70	7.50	7.29	6.96	6.53	2.68	3.56	3.32	7.80	7.41	7.74
H4-H5	7.32	7.48	6.81	0.31	1.12	1.13	1.65	6.89	4.71	7.49	7.59	7.40	0.59	1.00	1.13	7.10	6.26	5.88
H5-H6	1.19	6.96	6.73	3.71	6.28	8.26	1.94	2.53	7.56	2.46	7.04	6.13	2.15	2.77	8.29	1.27	2.76	7.50
H5-H7	2.71	2.91	3.33	1.40	8.86	3.21	2.07	7.15	2.28	1.90	2.91	1.91	2.66	8.30	3.25	2.86	7.39	2.39

Supplementary Table 14. $^3J_{H\text{-}H}$ (Hz) calculated by GIAO approach (B3LYP/6-311++G(2d,p)) for GlcNAc(6S) and GlcNAc(46S)

Atomic pair	GlcNAc(6S)									M-GlcNAc(6S)								
	$^4C_1$			$^1C_4$			$^2S_0$			$^4C_1$			$^1C_4$			$^2S_0$		
	gg	gt	tg	gg	gt	tg	gg	gt	tg	gg	gt	tg	gg	gt	tg	gg	gt	tg
H1-H2	5.14	5.74	5.52	1.98	1.58	1.08	3.01	0.13	2.00	6.36	6.08	5.99	1.84	1.59	1.11	2.27	0.14	0.01
H2-H3	7.61	7.42	8.08	2.58	2.59	2.05	8.99	3.42	8.41	7.37	7.84	6.07	2.62	2.56	2.08	0.74	2.64	4.63
H2-H8	0.05	0.16	1.11	6.92	7.11	7.17	0.47	2.04	0.21	7.21	4.23	0.02	6.79	6.48	7.17	5.78	2.88	8.49
H3-H4	6.85	6.07	6.13	2.37	2.93	2.82	4.72	6.95	6.51	6.53	5.92	6.10	2.50	2.97	2.84	3.11	6.93	7.48
H4-H5	6.62	7.41	6.44	0.23	0.59	1.54	0.57	5.97	2.44	6.86	7.42	6.76	0.34	0.59	1.54	7.90	7.17	5.16
H5-H6	2.88	4.60	7.73	2.06	2.15	8.51	0.62	8.32	2.36	1.17	5.65	5.51	1.51	1.92	8.51	2.66	6.54	2.38
H5-H7	1.31	0.54	4.39	2.37	7.84	3.99	5.00	3.33	7.78	3.10	1.06	5.47	3.11	7.68	4.04	1.79	1.31	7.88

Atomic pair	GlcNAc(46S)									M-GlcNAc(46S)								
	$^4C_1$			$^1C_4$			$^2S_0$			$^4C_1$			$^1C_4$			$^2S_0$		
	gg	gt	tg	gg	gt	tg	gg	gt	tg	gg	gt	tg	gg	gt	tg	gg	gt	tg
H1-H2	5.14	5.85	5.76	1.95	1.73	1.32	3.13	4.64	4.50	6.74	6.67	6.46	1.87	1.69	1.33	5.05	4.72	0.74
H2-H3	7.85	8.03	8.09	2.61	3.37	2.35	8.55	9.02	9.08	8.05	8.00	7.65	2.62	3.35	2.37	8.99	9.07	6.93
H2-H8	0.14	0.10	0.58	7.13	3.86	0.25	0.92	8.57	0.07	4.88	5.74	6.96	6.40	4.04	0.29	6.23	8.70	0.16
H3-H4	7.27	6.74	5.60	2.36	3.30	3.23	2.82	5.06	5.05	7.22	6.71	6.16	2.90	3.33	3.24	4.58	5.11	7.62
H4-H5	7.48	7.88	6.54	0.46	0.68	1.93	1.03	1.10	0.58	8.16	7.94	6.98	0.79	0.70	1.94	0.88	1.16	4.72
H5-H6	3.40	3.05	7.82	3.48	2.57	9.21	0.58	8.94	0.58	1.13	2.71	7.56	1.49	2.69	9.23	0.73	8.90	4.67
H5-H7	1.09	8.06	3.50	1.91	8.61	4.61	6.21	2.66	3.10	4.03	7.72	2.87	3.38	8.97	4.65	4.61	2.65	8.43

Supplementary Table 15. $^3J_{H\text{-}H}$ (Hz) calculated by GIAO approach (B3LYP/6-311++G(2d,p)) for GlcNAc and GalNAc(4S)

Atomic pair	GalNAc									M-GalNAc								
	$^4C_1$			$^1C_4$			$^2S_0$			$^4C_1$			$^1C_4$			$^2S_0$		
	gg	gt	tg	gg	gt	tg	gg	gt	tg	gg	gt	tg	gg	gt	tg	gg	gt	tg
H1-H2	5.68	5.70	5.53	1.99	1.46	1.17	2.75	3.37	2.03	5.73	5.33	6.19	1.29	1.46	1.27	5.14	0.14	0.15
H2-H3	7.52	7.66	7.87	3.07	2.94	3.14	8.22	8.42	8.04	7.82	7.54	7.84	3.07	3.21	3.16	8.74	4.32	4.29
H2-H8	0.86	1.08	0.88	6.44	7.17	8.81	0.17	0.10	7.27	1.58	1.10	8.20	7.37	7.11	9.15	3.06	3.73	3.61
H3-H4	3.14	3.08	3.90	4.56	3.70	2.61	3.95	4.94	3.57	2.61	3.22	3.72	3.95	3.45	2.57	4.74	2.94	3.31
H4-H5	1.91	1.49	2.08	7.28	6.07	5.23	7.31	6.55	6.51	1.43	1.23	1.88	6.88	6.49	5.23	7.31	5.14	5.50
H5-H6	1.00	6.96	6.40	3.13	8.19	3.74	1.65	8.32	4.45	1.25	5.87	6.49	1.77	8.43	3.95	3.02	7.16	5.26
H5-H7	2.81	2.60	5.65	1.43	6.30	8.37	2.08	4.39	7.88	2.64	1.23	5.49	2.78	3.23	8.41	1.10	2.68	7.78
Atomic pair	GalNAc(4S)									M-GalNAc(4S)								
	$^4C_1$			$^1C_4$			$^2S_0$			$^4C_1$			$^1C_4$			$^2S_0$		
	gg	gt	tg	gg	gt	tg	gg	gt	tg	gg	gt	tg	gg	gt	tg	gg	gt	tg
H1-H2	4.78	5.49	5.27	1.11	0.82	1.10	2.92	2.05	2.81	6.13	5.54	5.79	1.34	0.75	1.10	0.60	0.76	3.02
H2-H3	7.24	8.06	8.50	2.82	2.55	2.06	8.48	8.16	8.23	7.81	8.23	8.21	2.56	2.82	2.06	7.22	7.58	8.25
H2-H8	0.47	0.58	6.61	5.90	7.60	4.05	0.39	4.37	7.32	7.00	6.81	7.07	8.16	7.86	4.22	0.12	0.09	7.69
H3-H4	2.74	2.71	3.71	3.81	3.43	3.70	2.95	0.89	1.34	2.98	3.57	3.68	4.89	3.08	3.68	1.50	2.02	1.41
H4-H5	0.83	1.92	2.85	6.40	5.46	5.63	7.12	3.80	4.08	0.98	2.48	2.89	6.63	5.75	5.58	4.41	5.70	4.13
H5-H6	1.56	2.71	6.84	2.66	8.55	6.09	3.03	1.48	7.42	1.65	2.49	6.83	1.01	7.79	6.03	4.69	2.69	7.51
H5-H7	2.88	6.74	4.90	1.93	5.13	1.20	1.35	6.01	5.11	2.88	6.55	4.93	3.80	2.67	1.15	0.38	7.03	5.13

Supplementary Table 16. $^{3}J_{H\text{-}H}$ (Hz) calculated by GIAO approach (B3LYP/6-311++G(2d,p)) for GlcNAc(6S) and GlcNAc(46S)

Atomic pair	GalNAc(6S)									M-GalNAc(6S)								
	$^{4}C_{1}$			$^{1}C_{4}$			$^{2}S_{0}$			$^{4}C_{1}$			$^{1}C_{4}$			$^{2}S_{0}$		
	gg	gt	tg	gg	gt	tg	gg	gt	tg	gg	gt	tg	gg	gt	tg	gg	gt	tg
H1-H2	5.24	5.82	5.63	1.13	1.55	0.86	3.88	3.02	2.55	6.31	6.93	6.29	1.36	1.47	0.93	5.57	5.83	0.78
H2-H3	7.23	7.33	7.69	3.44	3.25	3.31	7.46	8.05	8.10	7.16	7.18	7.62	3.16	3.22	3.32	7.46	8.48	7.00
H2-H8	0.08	0.22	0.83	5.97	6.66	6.80	0.65	3.60	0.20	7.87	7.14	7.89	8.08	7.73	7.00	8.14	3.39	0.13
H3-H4	2.86	3.15	3.73	3.75	3.58	2.37	6.98	3.77	3.90	2.67	3.19	3.61	3.72	3.31	2.39	6.96	5.63	3.19
H4-H5	1.78	1.79	1.94	6.59	6.88	4.99	6.33	7.02	6.30	1.38	1.66	1.81	6.93	6.73	5.03	6.40	6.89	5.46
H5-H6	1.93	4.67	7.89	2.07	2.47	8.39	1.13	9.06	2.22	1.47	3.06	7.88	1.98	2.06	8.41	1.12	9.55	3.09
H5-H7	2.35	0.64	4.14	2.50	8.19	3.37	4.39	3.87	7.52	2.94	0.27	4.18	2.86	7.88	3.40	4.41	5.48	8.26
Atomic pair	GalNAc(46S)									M-GalNAc(46S)								
	$^{4}C_{1}$			$^{1}C_{4}$			$^{2}S_{0}$			$^{4}C_{1}$			$^{1}C_{4}$			$^{2}S_{0}$		
	gg	gt	tg	gg	gt	tg	gg	gt	tg	gg	gt	tg	gg	gt	tg	gg	gt	tg
H1-H2	5.38	5.49	5.39	1.31	1.60	1.37	4.96	5.65	0.63	6.35	6.23	6.07	1.55	1.48	1.43	7.09	4.04	1.61
H2-H3	7.57	7.99	8.43	2.47	3.35	3.55	8.19	8.45	8.07	7.36	8.02	8.40	3.79	3.47	3.65	7.32	8.56	7.84
H2-H8	0.14	0.21	0.64	8.94	7.25	6.05	4.17	5.24	0.38	6.32	2.50	7.14	0.77	7.91	6.06	10.48	5.82	0.02
H3-H4	2.90	1.70	3.36	4.76	4.00	2.89	3.00	7.28	1.40	2.87	1.72	3.34	4.26	3.56	2.86	3.15	5.49	2.97
H4-H5	2.21	1.58	2.97	7.59	7.19	5.95	6.93	7.42	4.95	1.84	1.39	2.81	7.08	6.90	5.95	6.43	7.79	6.96
H5-H6	2.26	6.63	6.23	1.86	3.58	7.97	3.94	9.16	7.09	1.71	7.47	6.14	0.94	2.94	8.12	4.13	7.30	4.45
H5-H7	2.53	6.20	5.98	3.42	9.18	2.23	1.04	3.80	7.08	3.12	5.84	5.90	4.46	8.82	2.37	0.93	2.19	8.37

Supplementary Table 17. $^{3}J_{H\text{-}H}$ (Hz) calculated by GIAO approach (B3LYP/6-311++G(2d,p)) for GlcUA and IdoUA derivatives

Atomic pair	GlcUA			M-GlcUA			IdoUA			M-IdoUA		
	$^{4}C_{1}$	$^{1}C_{4}$	$^{2}S_{0}$	$^{4}C_{1}$	$^{1}C_{4}$	$^{2}S_{0}$	$^{4}C_{1}$	$^{1}C_{4}$	$^{2}S_{0}$	$^{4}C_{1}$	$^{1}C_{4}$	$^{2}S_{0}$
H1-H2	4.81	1.69	4.26	5.45	0.47	3.30	5.01	1.08	2.94	5.14	1.17	3.04
H2-H3	6.25	2.87	8.01	6.32	2.36	7.57	6.12	2.23	7.62	6.20	2.26	7.67
H3-H4	6.00	3.01	3.85	5.97	3.67	4.16	7.03	3.01	2.51	7.11	2.94	2.51
H4-H5	6.94	1.10	0.29	6.96	2.36	0.04	5.64	1.38	3.24	5.54	1.42	3.32
Atomic pair	GlcUA(2S)			M-GlcUA(2S)			IdoUA(2S)			M-IdoUA(2S)		
	$^{4}C_{1}$	$^{1}C_{4}$	$^{2}S_{0}$	$^{4}C_{1}$	$^{1}C_{4}$	$^{2}S_{0}$	$^{4}C_{1}$	$^{1}C_{4}$	$^{2}S_{0}$	$^{4}C_{1}$	$^{1}C_{4}$	$^{2}S_{0}$
H1-H2	4.82	0.82	3.46	5.22	1.69	4.05	5.61	1.12	2.94	5.24	1.70	3.01
H2-H3	7.00	2.66	8.00	6.64	3.13	8.86	7.44	2.17	7.60	6.38	2.87	7.55
H3-H4	6.40	3.68	5.43	6.55	2.99	4.67	6.91	2.40	2.61	7.23	2.91	2.87
H4-H5	7.08	2.69	1.02	7.08	1.84	0.16	5.70	1.75	3.12	5.48	2.13	3.40
Atomic pair	GlcUA(3S)			M-GlcUA(3S)			IdoUA(3S)			M-IdoUA(3S)		
	$^{4}C_{1}$	$^{1}C_{4}$	$^{2}S_{0}$	$^{4}C_{1}$	$^{1}C_{4}$	$^{2}S_{0}$	$^{4}C_{1}$	$^{1}C_{4}$	$^{2}S_{0}$	$^{4}C_{1}$	$^{1}C_{4}$	$^{2}S_{0}$
H1-H2	5.16	0.88	4.47	5.54	0.63	3.98	3.83	1.11	2.27	4.23	1.18	2.40
H2-H3	5.67	2.74	8.85	5.24	2.75	8.82	7.71	2.49	7.60	7.62	2.50	7.71
H3-H4	5.41	3.56	2.70	5.39	3.93	4.14	8.34	3.49	3.53	8.45	3.45	3.59
H4-H5	7.07	2.04	0.51	7.20	1.98	0.70	4.74	1.49	3.65	4.72	1.50	3.75
Atomic pair	GlcUA(23S)			M-GlcUA(23S)			IdoUA(23S)			M-IdoUA(23S)		
	$^{4}C_{1}$	$^{1}C_{4}$	$^{2}S_{0}$	$^{4}C_{1}$	$^{1}C_{4}$	$^{2}S_{0}$	$^{4}C_{1}$	$^{1}C_{4}$	$^{2}S_{0}$	$^{4}C_{1}$	$^{1}C_{4}$	$^{2}S_{0}$
H1-H2	4.99	1.06	5.59	5.55	1.05	4.54	6.04	1.55	3.32	4.82	1.53	3.66
H2-H3	8.06	2.73	9.42	4.97	2.87	9.30	7.72	3.08	8.85	8.74	2.44	8.37
H3-H4	8.08	2.90	3.01	5.46	3.02	2.70	6.89	3.17	2.37	8.23	2.57	1.93
H4-H5	6.48	1.44	0.54	7.43	1.29	0.42	6.24	3.43	3.32	4.99	3.55	3.16

Supplementary Table 18. ^{1}H chemical shifts (ppm) in CH_3 of the acetyl group of Glc/GalNAc derivatives.

Monosaccharide	^{1}H chemical shifts, ppm	Monosaccharide	^{1}H chemical shifts, ppm
GlcNAc	2.047	GlcNAc	2.063
GlcNAc(4S)	2.048	GlcNAc(4S)	2.086
GlcNAc(6S)	2.089	GlcNAc(6S)	2.101
GlcNAc(46S)	2.096	GlcNAc(46S)	2.104
M-GlcNAc	1.968	M-GalNAc	1.990
M-GlcNAc(4S)	1.994	M-GalNAc(4S)	2.062
M-GlcNAc(6S)	2.071	M-GalNAc(6S)	2.038
M-GlcNAc(46S)	2.031	M-GalNAc(46S)	2.043

Chemical shifts presented are mean values for all gg/gt/tg conformations of the monosaccharide in $^{4}C_1$ ring conformation.

Supplementary Table 19. [1]H chemical shifts (ppm) in CH_3 of the acetyl group of Glc/GalNAc derivatives for gg/gt/tg conformations

Molekule	Proton	ppm, gg	ppm, gt	ppm, tg
GlcNAc	H9	1.70	1.69	1.70
	H10	2.21	2.22	2.21
	H11	2.25	2.23	2.21
GlcNAc(4S)	H9	1.68	1.68	1.76
	H10	2.16	2.19	2.24
	H11	2.21	2.21	2.30
GlcNAc(6S)	H9	1.77	1.76	1.67
	H10	2.22	2.27	2.21
	H11	2.32	2.32	2.26
GlcNAc(46S)	H9	1.81	1.82	1.73
	H10	2.25	2.21	2.20
	H11	2.33	2.29	2.22
GalNAc	H9	1.74	1.73	1.72
	H10	2.23	2.23	2.18
	H11	2.24	2.26	2.24
GalNAc(4S)	H9	1.77	1.76	1.70
	H10	2.22	2.18	2.31
	H11	2.32	2.20	2.31
GalNAc(6S)	H9	1.82	1.81	1.71
	H10	2.26	2.31	2.16
	H11	2.32	2.32	2.20
GalNAc(46S)	H9	1.84	1.87	1.74
	H10	2.21	2.20	2.19
	H11	2.33	2.35	2.21
M-GlcNAc	H9	1.53	1.54	1.49

	H10	2.19	2.20	2.08
	H11	2.24	2.24	2.20
M-GlcNAc(4S)	H9	1.56	1.48	1.75
	H10	2.19	2.11	2.24
	H11	2.19	2.17	2.26
M-GlcNAc(6S)	H9	1.64	1.72	1.72
	H10	2.15	2.11	2.25
	H11	2.40	2.37	2.28
M-GlcNAc(46S)	H9	1.69	1.73	1.55
	H10	2.15	2.12	2.16
	H11	2.26	2.36	2.26
M-GalNAc	H9	1.60	1.57	1.55
	H10	2.17	2.20	2.18
	H11	2.20	2.21	2.23
M-GalNAc(4S)	H9	1.55	1.64	1.73
	H10	2.14	2.28	2.28
	H11	2.29	2.29	2.36
M-GalNAc(6S)	H9	1.63	1.65	1.55
	H10	2.18	2.21	2.13
	H11	2.38	2.38	2.23
M-GalNAc(46S)	H9	1.68	1.76	1.56
	H10	2.11	2.10	2.17
	H11	2.43	2.36	2.22

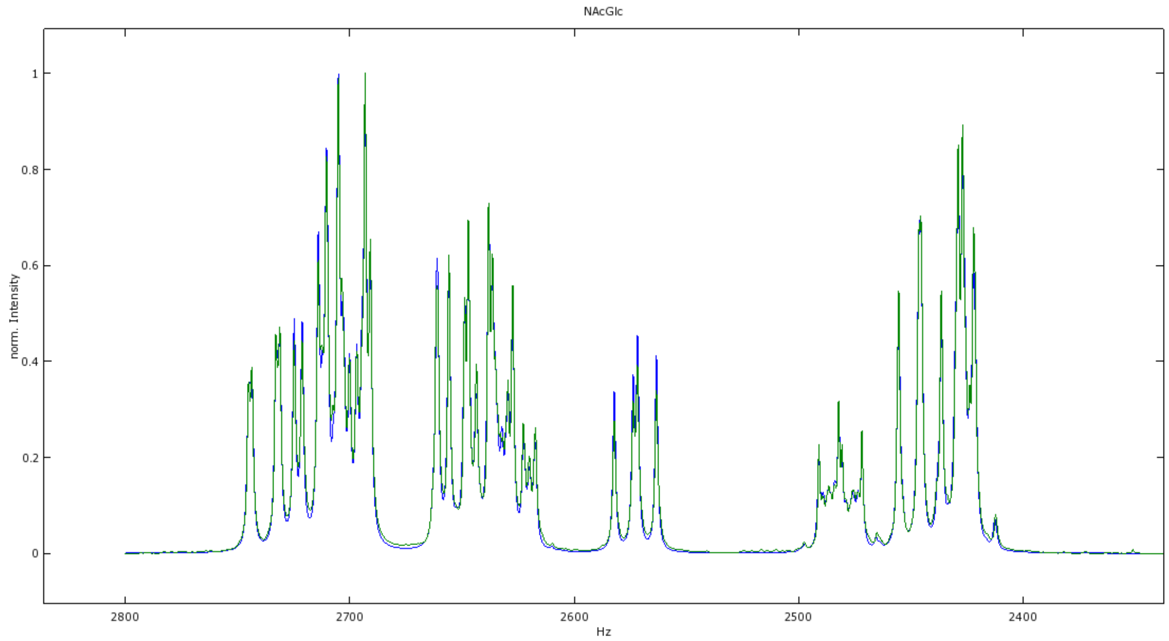

Supplementary Figure 1. Comparison of the simulated (blue) and measured NMR spectrum (green) of NAcGlc. The simulated spectra were calculated using a self-written Octave [43] script and simulating the alpha- and beta form by assuming a single line width per molecule. The signal intensities were normalized to the highest sugar peak in the present subpart of the spectrum.

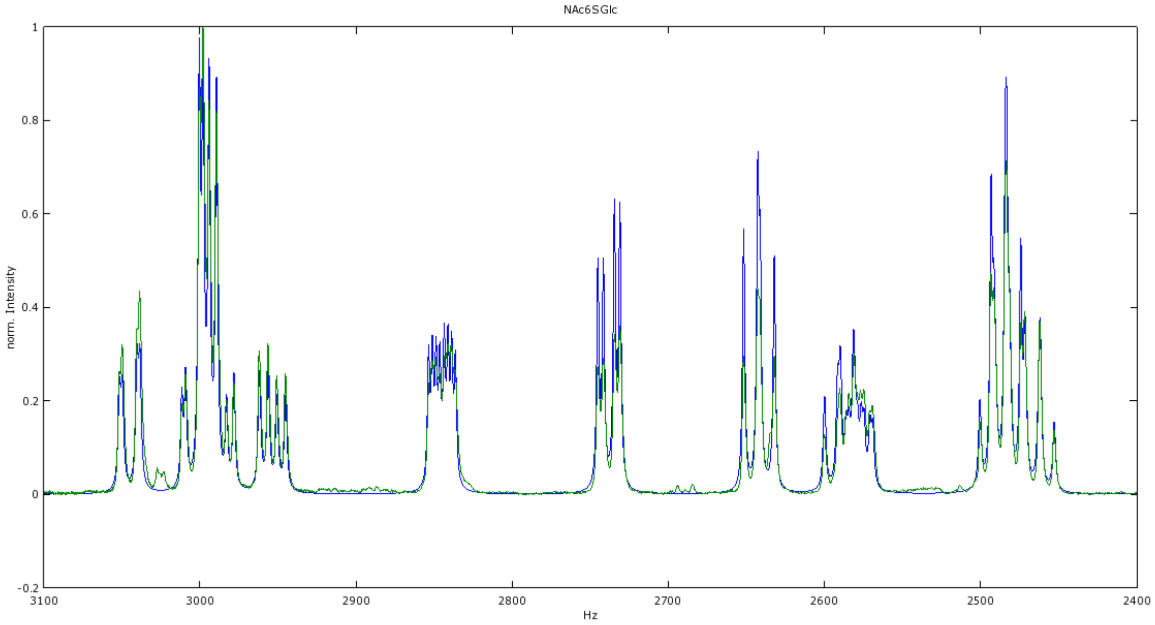

Supplementary Figure 2. Comparison of the simulated (blue) and measured NMR spectrum (green) of NAc6SGlc. The simulated spectra were calculated using a self-written Octave [43] script and simulating the alpha- and beta form by assuming a single line width per molecule. The signal intensities were normalized to the highest sugar peak in the present subpart of the spectrum.

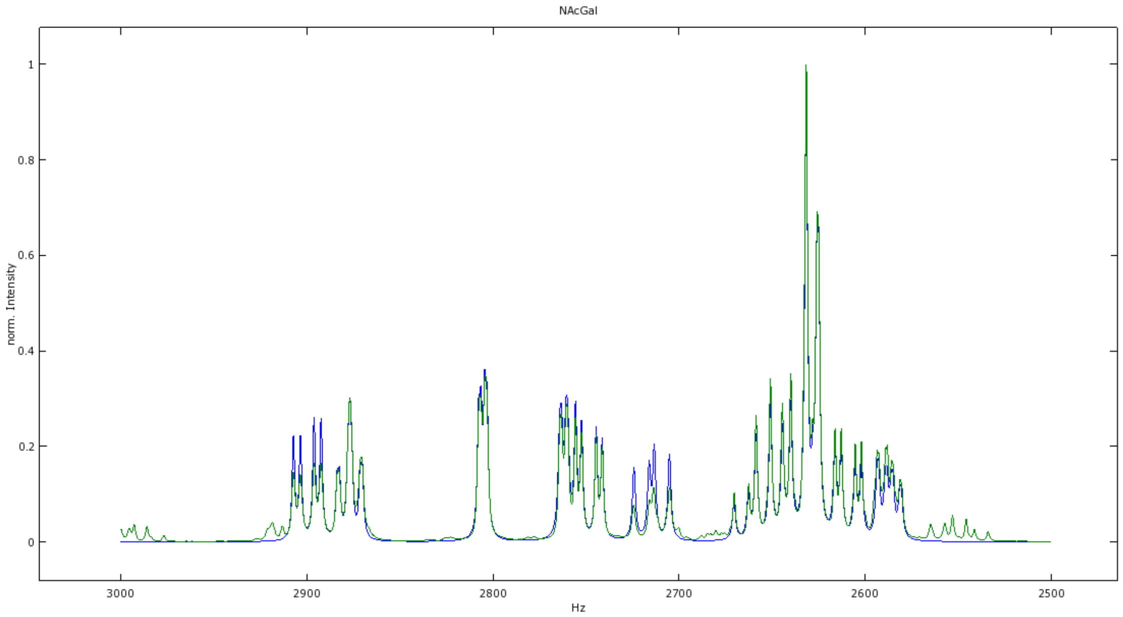

Supplementary Figure 3. Comparison of the simulated (blue) and measured NMR spectrum (green) of NAcGal. The simulated spectra were calculated using a self-written Octave [43] script and simulating the alpha- and beta form by assuming a single line width per molecule. The signal intensities were normalized to the highest sugar peak in the present subpart of the spectrum.

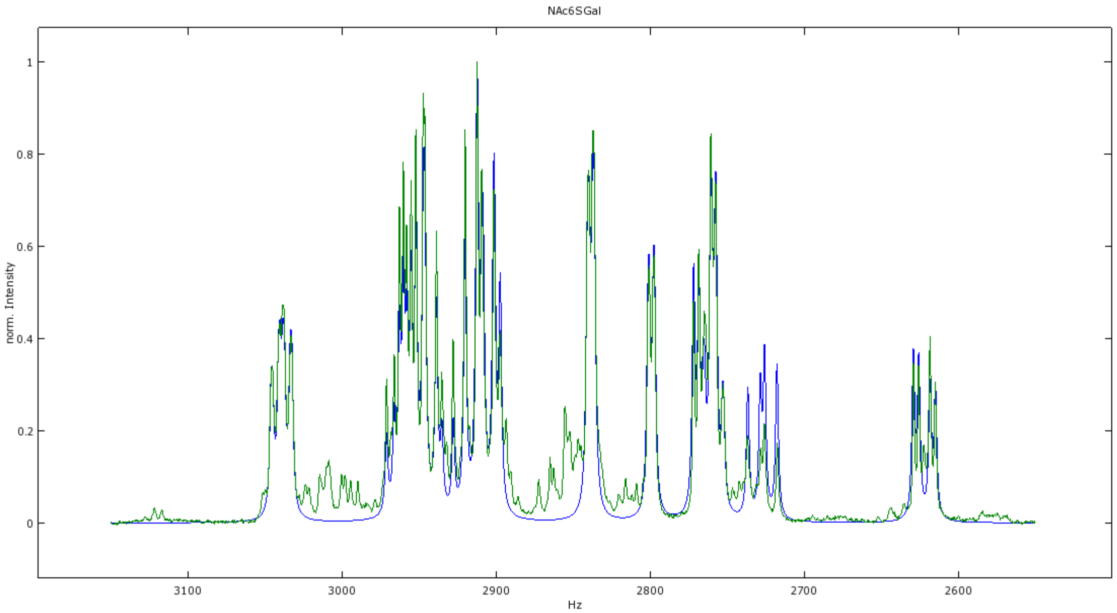

Supplementary Figure 4. Comparison of the simulated (blue) and measured NMR spectrum (green) of NAc6SGal. The simulated spectra were calculated using a self-written Octave [43] script and simulating the alpha- and beta form by assuming a single line width per molecule. The signal intensities were normalized to the highest sugar peak in the present subpart of the spectrum.

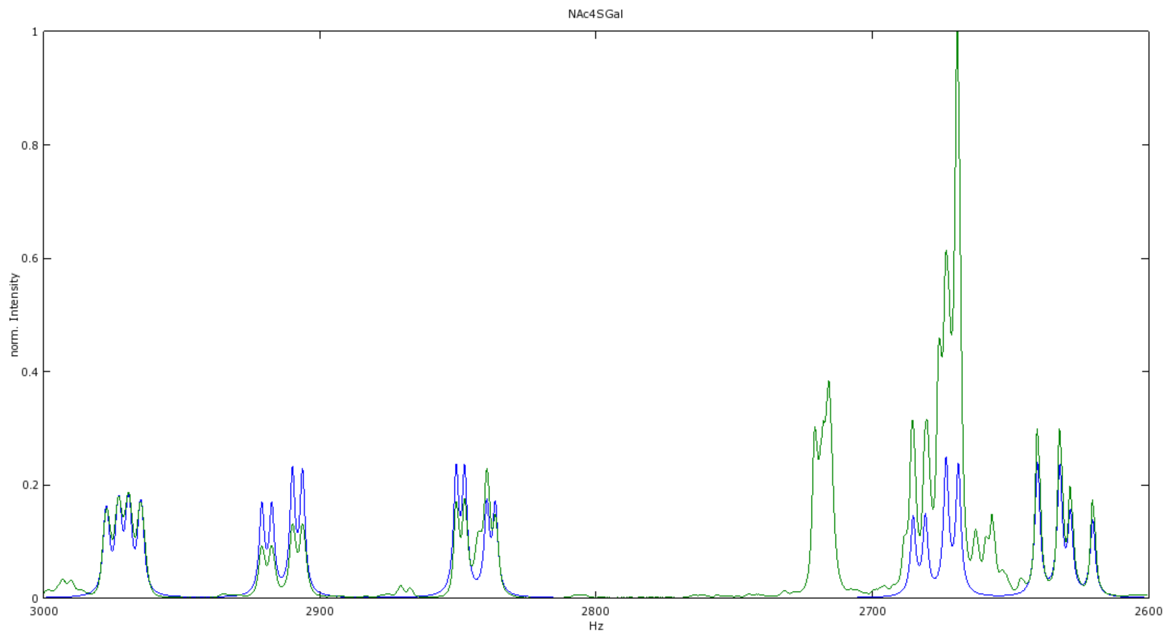

Supplementary Figure 5. Comparison of the simulated (blue) and measured NMR spectrum (green) of NAc4SGal. The simulated spectra were calculated using a self-written Octave [43] script and simulating the alpha form only by assuming a single line width per molecule. The signal intensities were normalized to the highest sugar peak in the present subpart of the spectrum.
